# Decanoic acid inhibits mTORC1 activity independent of glucose and insulin signaling

**DOI:** 10.1073/pnas.2008980117

**Published:** 2020-09-02

**Authors:** Eleanor C. Warren, Stephanie Dooves, Eleonora Lugarà, Joseph Damstra-Oddy, Judith Schaf, Vivi M. Heine, Mathew C. Walker, Robin S. B. Williams

**Affiliations:** ^a^Centre for Biomedical Sciences, Department of Biological Sciences, Royal Holloway University of London, Egham TW20 0EX, United Kingdom;; ^b^Department of Child and Youth Psychiatry, Amsterdam Universitair Medische Centra, Amsterdam Neuroscience, Vrije Universiteit Amsterdam, 1081 HV Amsterdam, The Netherlands;; ^c^Clinical and Experimental Epilepsy UCL, Queen Square Institute of Neurology, University College London, London WC1N 3BG, United Kingdom;; ^d^Department of Complex Trait Genetics, Centre for Neurogenomics and Cognitive Research, Amsterdam Neuroscience, Vrije Universiteit Amsterdam, 1081 HV Amsterdam, The Netherlands

**Keywords:** *Dictyostelium discoideum*, epilepsy, mTOR, decanoic acid, tuberous sclerosis complex

## Abstract

Low-glucose and -insulin conditions, associated with ketogenic diets, can reduce the activity of the mechanistic target of rapamycin complex 1 (mTORC1) signaling pathway, potentially leading to a range of positive medical and health-related effects. Here, we determined whether mTORC1 signaling is also a target for decanoic acid, a key component of the medium-chain triglyceride (MCT) ketogenic diet. Using a tractable model system, *Dictyostelium*, we show that decanoic acid can decrease mTORC1 activity, under conditions of constant glucose and in the absence of insulin, measured by phosphorylation of eukaryotic translation initiation factor 4E-binding protein 1 (4E-BP1). We determine that this effect of decanoic acid is dependent on a ubiquitin regulatory X domain-containing protein, mediating inhibition of a conserved *Dictyostelium* AAA ATPase, p97, a homolog of the human transitional endoplasmic reticulum ATPase (VCP/p97) protein. We then demonstrate that decanoic acid decreases mTORC1 activity in the absence of insulin and under high-glucose conditions in ex vivo rat hippocampus and in tuberous sclerosis complex (TSC) patient-derived astrocytes. Our data therefore indicate that dietary decanoic acid may provide a new therapeutic approach to down-regulate mTORC1 signaling.

## Significance

The mTORC1 complex provides a critical role in cell function, regulating a variety of processes including growth and autophagy. mTORC1 signaling is hyperactivated in a range of common diseases including cancer, epilepsy, and neurodegenerative disorders. Hence, mTORC1 signaling provides an important target for regulation in many contexts. Here, we show that decanoic acid, a key component of a widely used medicinal diet, reduces mTORC1 activity. We identify this in a tractable model system, and validate it in ex vivo rat brain tissue and in human iPSC-derived astrocytes from patients with a clinically relevant disease. Thus, we provide insight into an easily accessible therapeutic approach for a range of diseases.

Inhibition of mechanistic target of rapamycin complex 1 (mTORC1) has been suggested as a common mechanism contributing to the therapeutic benefits of ketogenic diets through a reduction in glucose and insulin (1–4). This inhibitory activity may provide a component of the therapeutic efficacy of these diets in the treatment of patients with drug-resistant epilepsies (5), cancers (6), and neurodegenerative disorders (7), and in providing lifespan extension (4). Inhibition of mTORC1 has been proposed to lead to increased autophagy and the clearance of misfolded proteins in neurodegenerative disorders (8), the decrease of cell proliferation in cancers (9), and reduced stress response and improved mitochondrial function credited with increasing longevity (10). Hyperactivation of mTORC1 activity is also found in the neurodevelopmental disorder tuberous sclerosis complex (TSC) (11), resulting from mutations in either the gene for hamartin (TSC1) or tuberin (TSC2), causing the development of tumors and manifesting in epilepsy, cognitive disability, and neurobehavioral abnormalities (12). The use of mTORC1 inhibitors to treat patients with TSC appears clinically promising (13, 14). mTORC1 signaling is also disrupted by mutations in p97 (also called VCP or cdcD, referred to as human p97; hp97), an evolutionarily conserved AAA ATPase (15), contributing to the pathogenesis of degenerative diseases (16). This critical ATPase functions in processes such as endoplasmic reticulum-associated degradation (ERAD) (17), autophagy (18), and DNA damage repair (19), with UBX domain-containing proteins making up the largest group of cofactors required for p97 function (20).

Ketogenic diets were initially developed to mimic starvation, with the low-carbohydrate and high-fat intake leading to the generation of ketone bodies (21). The initial form of the diet, termed the “classical” ketogenic diet, requires 90% of dietary energy to be delivered through fats and, despite its efficacy, is difficult to maintain due to stringent dietary restrictions (22). To reduce these restrictions, a modified form of the diet, called the medium-chain triglyceride (MCT) ketogenic diet, was introduced in 1971 (23), requiring around 30% less dietary energy delivered as fats, with improved tolerability (24, 25). Metabolic breakdown of these dietary triglycerides in the intestine leads to the release of the fatty acids octanoic acid (C8) and decanoic acid (C10), which are absorbed and transported in the blood to the brain, where they cross the blood–brain barrier (26). Recently, it has been suggested that ketosis may not be necessary for an antiseizure effect of the MCT diet and that decanoic acid alone can mediate an antiseizure effect (21). Several mechanisms of action have been suggested for decanoic acid, including the direct inhibition of excitatory α-amino-3-hydroxy-5-methyl-4-isoxazolepropionic acid (AMPA) receptors (5, 27), the activation of the nuclear peroxisome proliferator-activated receptor gamma receptor leading to increased mitochondrial proliferation (28), as well as the inhibition of phosphoinositide turnover and the inhibition of diacylglycerol kinase (29, 30). However, a potential disadvantage of nonketogenic MCT diets is a lack of effect on glucose and insulin and therefore mTORC1 signaling, but this has not been previously investigated. We therefore wished to test this hypothesis that decanoic acid has no effect on mTORC1 activity.

The eukaryotic social amoeba *Dictyostelium discoideum* is a simple model organism which provides a valuable system for biomedical research. *Dictyostelium* has a well-described life cycle where it exists in both single and multicellular stages (31). Many *Dictyostelium* proteins are more homologous to human proteins than to those of unicellular fungi, making it a valuable organism for studying processes relevant to humans (32). The generation of libraries of *Dictyostelium* mutants has enabled pharmacogenetic studies leading to the identification of targets for compounds (33, 34). This approach allows rapid identification of proteins that control sensitivity to a compound, thus implicating the proteins or the wider pathways in the action of the compound. In epilepsy research, *Dictyostelium* has been employed to identify a mechanism of the commonly used antiepileptic drug valproate to reduce phosphoinositide signaling (30), with more potent compounds showing improved efficacy in seizure models (30, 35, 36). Through this research originating in *Dictyostelium*, decanoic acid has been identified as an effective seizure control agent (30, 37), contributing to the development and progression to clinical trials of an MCT diet with an altered decanoic acid content (NCT02825745; https://clinicaltrials.gov/ct2/show/NCT02825745).

In this study, we investigate a role for decanoic acid in mTORC1 function. We initially show that decanoic acid causes a reduction of mTORC1 activity in a structurally specific manner in *Dictyostelium*, in the absence of insulin and in the presence of glucose, suggesting a specific molecular mechanism for decanoic acid in regulating this pathway. To identify this mechanism, we screened a mutant library to identify a decanoic acid-resistant mutant lacking a UBX domain-containing protein (UBXD18) that is partially resistant to the effects of decanoic acid on growth and mTORC1 signaling. We establish that UBXD18 binds the *Dictyostelium* p97 protein, decanoic acid reduces the activity of *Dictyostelium* p97 in a UBXD18-dependent manner, and p97 inhibition is sufficient to cause a reduction in mTORC1 signaling in this model. We then translate our findings to a rat hippocampal slice model and astrocytes derived from patients with the neurodevelopmental disorder TSC, to demonstrate that decanoic acid also reduces normal and dysregulated mTORC1 signaling in mammalian systems.

## Results

### Decanoic Acid Reduces mTORC1 Signaling in *Dictyostelium*.

**Fig. 1.**
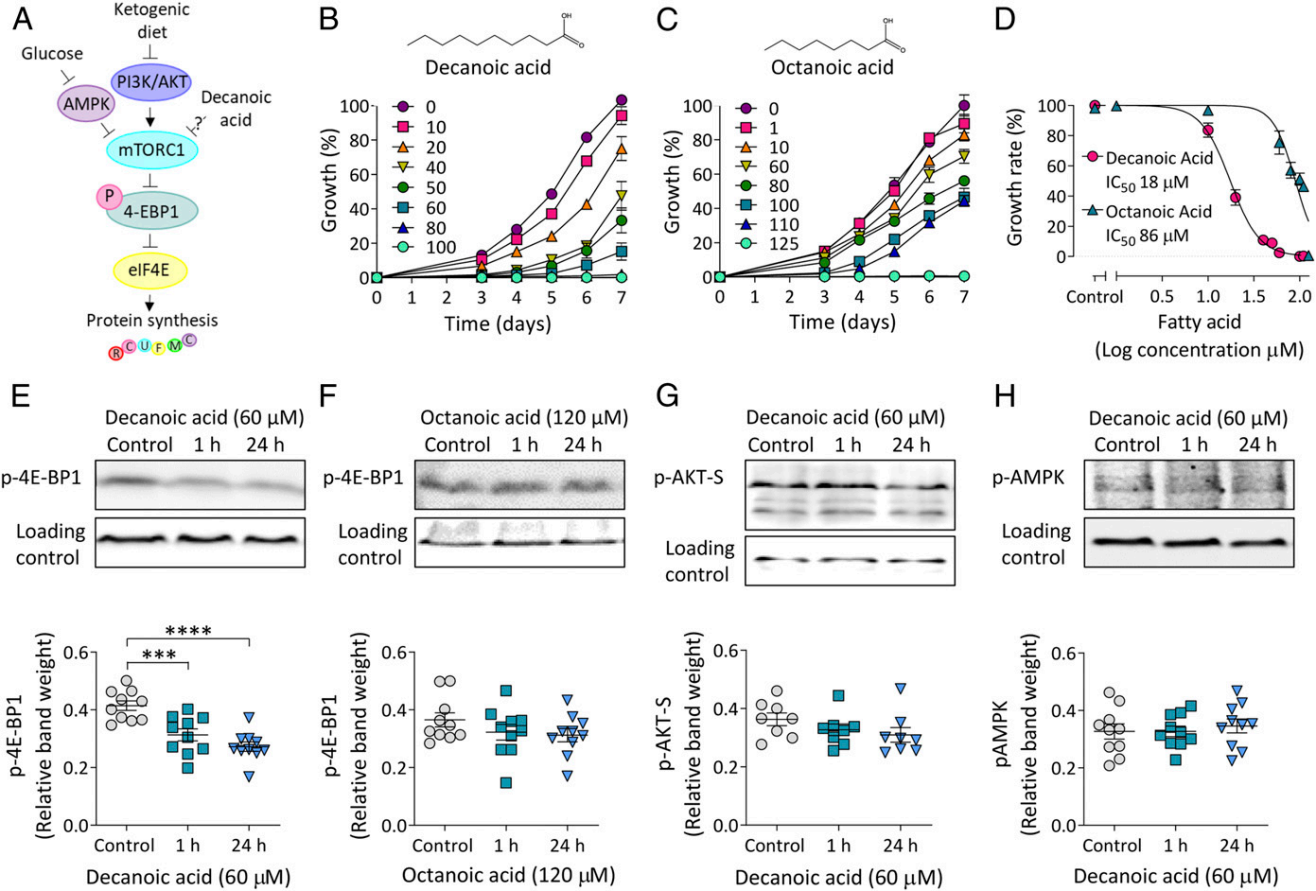
Decanoic acid causes a reduction in p-4E-BP1 levels in *Dictyostelium*. (*A*) Simplified schematic diagram of mTORC1 pathway signaling. Glucose activates mTORC1 through inhibition of AMPK. Conversely, the ketogenic diet inhibits mTORC1 through inhibition of PI3K/AKT signaling. Phosphorylation of 4E-BP1 by mTORC1 induces 4E-BP1 to dissociate from eukaryotic translation initiation factor 4E (eIF4E), promoting the initiation of translation, and this phosphorylation is used in this study as a readout for mTORC1 activation. (*B* and *C*) *Dictyostelium* cells were treated with (*B*) decanoic acid (*n* = 12) or (*C*) octanoic acid (*n* = 6) for 7 d at a range of concentrations (µM). Percentage growth was plotted normalized to the solvent control (0 µM, 0.2% DMSO) at 7 d. (*D*) Dose–response curves of normalized growth rate plotted against log concentration of compound were used to calculate IC_50_ values. (*E* and *F*) p-4E-BP1 levels were analyzed in wild-type cells treated for 1 or 24 h with (*E*) decanoic acid (*n* = 10) (one-way ANOVA with Dunnett’s post hoc test) or (*F*) octanoic acid (*n* = 10) (one-way ANOVA with Dunnett’s post hoc test) at concentrations corresponding to an ∼95% reduction in growth rate. Methylcrotonyl-CoA carboxylase (MCCC1) served as a loading control. (*G*) AKT activity was evaluated in wild-type cells treated for 1 or 24 h with decanoic acid using an anti–phospho-AKT substrate antibody, with MCCC1 as a loading control (*n* = 10) (one-way ANOVA with Dunnett’s post hoc test). (*H*) AMPK activation was analyzed using an antibody against phospho-AMPK, with MCCC1 as a loading control (*n* = 10) (one-way ANOVA with Dunnett’s post hoc test). Data represent the mean ± SEM. Significance is indicated by ***P ≤ 0.001, ****P ≤ 0.0001.

As wide-ranging therapeutic benefits of the classical ketogenic diet have been associated with inhibition of mTORC1 signaling (1, 4, 8, 9) (Fig. 1*A*), we employed *Dictyostelium* to investigate the effects of the MCT diet constituents decanoic acid and octanoic acid on this activity. We initially assessed the effects of these medium-chain fatty acids on *Dictyostelium* unicellular growth and multicellular development to establish relevant concentrations of decanoic acid and octanoic acid to investigate in this model. *Dictyostelium* cell growth was quantified in the presence of a range of concentrations of both fatty acids, with cell counts being recorded once every 24 h from 3 to 7 d posttreatment (Fig. 1 *B* and *C*). Treatment with both fatty acids caused a dose-dependent inhibition of growth. The rate of the exponential growth phase was then used to plot nonlinear regression curves, and half-maximal inhibitory concentrations (IC_50_ values) were calculated. Decanoic acid provided an IC_50_ of 18 µM (Fig. 1 *B* and *D*), while octanoic acid was less potent with an IC_50_ of 86 µM (Fig. 1 *C* and *D*). Since *Dictyostelium* also show a multicellular development cycle, we also investigated these fatty acids in regulating this process, where starving cells form fruiting bodies comprising dead vacuolated stalks holding spore heads aloft under control condition cells (*SI Appendix*, Fig. S1). This developmental process was unaffected by decanoic acid and octanoic acid at concentrations of fatty acids (200 µM) far greater than that shown to inhibit cell growth, suggesting that these compounds regulate molecular targets required for cell proliferation but not development (Fig. 1 *B*–*D* and *SI Appendix*, Fig. S1). These data also suggest that the medium-chain fatty acids have a specific molecular effect on *Dictyostelium* rather than a general toxic effect.

In order to monitor the effects of decanoic acid and octanoic acid on mTORC1 signaling in *Dictyostelium*, we quantified levels of phosphorylated 4E-BP1 (p-4E-BP1), a translation repressor directly phosphorylated by mTORC1 (Fig. 1*A*) (38, 39). Concentrations of decanoic acid and octanoic acid corresponding to an ∼95% reduction in growth rate (60 and 120 µM, respectively) (Fig. 1*D*) were employed, providing concentrations relevant to those observed in plasma of patients on the MCT diet (decanoic acid, 157 µM; octanoic acid, 310 µM) (24, 40). *Dictyostelium* cells were treated with fatty acids for 1 or 24 h before p-4E-BP1 levels were quantified by Western blot (Fig. 1 *E* and *F*). Decanoic acid decreased p-4E-BP1 levels at 1 and 24 h (Fig. 1*E*), while treatment with octanoic acid had no significant effect on the levels of p-4E-BP1 at either treatment duration (Fig. 1*F*). Starvation, a physiological process well-established to reduce mTORC1 activity (41, 42), also caused a significant decrease in p-4E-BP1 levels in *Dictyostelium* (*SI Appendix*, Fig. S2*A*), as did two established mTOR inhibitors (38, 43) (*SI Appendix*, Fig. S2 *B* and *C*). Rapamycin did not significantly alter p-4E-BP1 levels under our conditions (*SI Appendix*, Fig. S2*D*), consistent with findings showing a lack of autophagic response to rapamycin in *Dictyostelium* (44).

Since mTORC1 is activated by phosphatidylinositol 3-kinase (PI3K)/AKT signaling and inhibited by AMP-activated protein kinase (AMPK) signaling (Fig. 1*A*), we also investigated a role for decanoic acid in these signaling pathways. We monitored the effects of decanoic acid on PI3K/AKT signaling by measuring levels of a phosphorylated AKT substrate (p-AKT-S) (45, 46) (*SI Appendix*, Fig. S2 *E* and *F*). No significant difference was observed in p-AKT-S levels after treatment with decanoic acid (60 µM) for 1 or 24 h (Fig. 1*G*). This indicated that decanoic acid targets mTORC1 independent of PI3K/AKT signaling under these conditions, and further suggests that decanoic acid inhibits mTORC1 without altering mTORC2 activation. In these growing cells, it was not possible to assay PI3K signaling through monitoring phosphorylation of the activation loops of the AKT homologs PKBR1 (T309) and PKBA (T278), since this signaling is undetectable during growth (45, 47) (*SI Appendix*, Fig. S2*G*). We also evaluated the effect of decanoic acid on AMPK signaling by assessing phospho-AMPKα (p-AMPK) levels (39, 48) (*SI Appendix*, Fig. S2 *H*–*K*), where no significant difference was observed (Fig. 1*H*).

### Identification of UBXD18 as a Potential Molecular Target for Decanoic Acid.

**Fig. 2.**
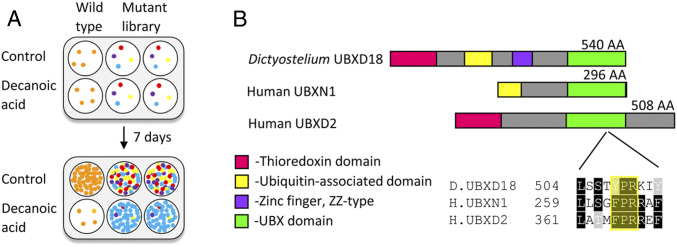
*Dictyostelium* UBXD18 protein, identified from a mutant library screen to regulate decanoic acid sensitivity, contains the evolutionarily conserved UBX domain. (*A*) A genetic screen of an insertional mutant library was carried out to identify mutants partially resistant to the growth-inhibitory effects of medium-chain fatty acids. (*B*) The genetic screen identified a gene encoding UBX domain-containing protein 18 (DDB_G0276057) as being a potential target for decanoic acid. The two most highly conserved human proteins, UBXN1 and UBXD2, are shown alongside a schematic of the *Dictyostelium* UBXD18 protein. These proteins share a common UBX domain with a highly conserved s3/s4 loop involved in binding p97 (highlighted).

We sought to identify potential molecular targets for decanoic acid by isolating decanoic acid-resistant mutants using an unbiased genetic screen of a library of *Dictyostelium* insertional mutants (Fig. 2*A* and *SI Appendix*, Fig. S3*A*). The library of mutants was screened with a concentration of decanoic acid that inhibits wild-type cell growth (120 µM; 7 d). Using this approach, mutants resistant to decanoic acid were isolated, and the location of the insertion site within the genome of these mutants was determined to identify genes controlling decanoic acid sensitivity. From this, a decanoic acid-resistant mutant, *UBXD18*^*−*^, was identified (Fig. 2*B*), with the mutagenic insertion in the region coding for the ubiquitin-associated domain of the *ubxd18* gene (DDB_G0276057).

Proteomic analysis enabled the initial characterization of the *Dictyostelium* UBXD18 protein. The UBXD18 protein sequence shared homology with two human UBX domain-containing proteins (UBXN1, UniProt Q04323; UBXD2, UniProt Q92575) (Fig. 2*B*), with highly conserved UBX domains (*SI Appendix*, Fig. S3*B*). The *Dictyostelium* UBX domain shared 28% identical and 51% similar amino acids with the UBX domain of UBXN1, and 27% identical and 51% similar amino acids with the UBX domain of UBXD2 (*SI Appendix*, Fig. S3*B*). A binding loop (s3/s4) known to interact with the AAA ATPase p97 was partially conserved between these proteins (*SI Appendix*, Fig. S3*B*) (49), suggesting that these proteins are p97-binding partners. Cladistic analysis of all *Dictyostelium* and human UBX proteins (*SI Appendix*, Fig. S3*C*) confirmed that the UBXN1 and UBXD2 proteins are the most likely homologs of the *Dictyostelium* UBXD18 protein.

### The Effects of Decanoic Acid on *Dictyostelium* Growth and mTORC1 Signaling Are Dependent on UBXD18.

**Fig. 3.**
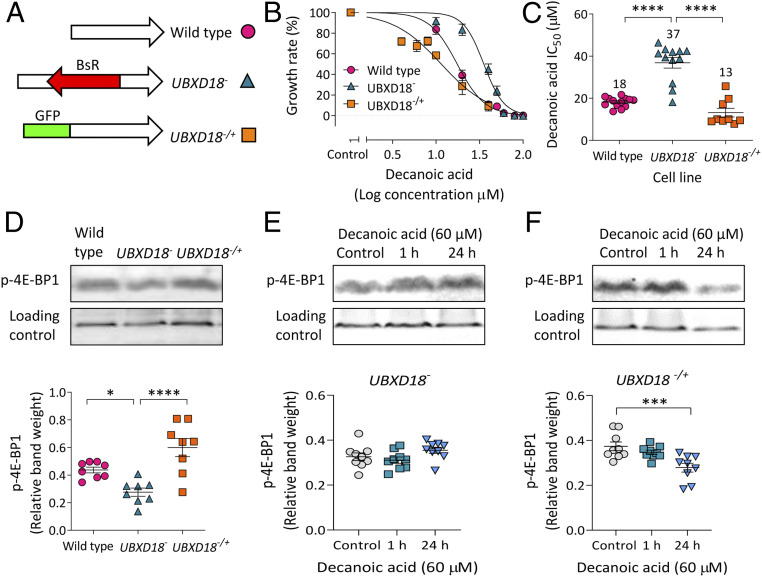
UBXD18 regulates the sensitivity of *Dictyostelium* to decanoic acid. (*A*) A UBXD18 knockout (*UBXD18*^*−*^) was generated by inserting a blasticidin resistance sequence (BsR) into the *ubxd18* gene, and a rescue (*UBXD18*^*−/+*^) was generated by expressing the *ubxd18* gene attached to GFP. (*B*) Growth analysis of wild-type (*n* = 12), *UBXD18*^*−*^ (*n* = 12), and *UBXD18*^−/+^ (*n* = 9) *Dictyostelium* cells in decanoic acid, displayed as dose–response curves of normalized cell density plotted against log concentration. (*C*) IC_50_ values were compared between wild type (*n* = 12), *UBXD18*^*−*^ (*n* = 12), and *UBXD18*^*−/+*^ (*n* = 9) (one-way ANOVA with Dunnett’s post hoc test). (*D*) Analysis of p-4E-BP1 levels in wild type, *UBXD18*^*−*^, and *UBXD18*^*−/+*^, with MCCC1 as a loading control (*n* = 8) (one-way ANOVA with Dunnett’s post hoc test). (*E* and *F*) Analysis of p-4E-BP1 levels in (*E*) *UBXD18*^*−*^ (*n* = 9) (Kruskal–Wallis test with Dunn’s post hoc test) or (*F*) *UBXD18*^*−/+*^ (*n* = 9) (one-way ANOVA with Dunnett’s post hoc test) treated with decanoic acid (60 µM) for 1 or 24 h, with MCCC1 as a loading control. Data represent the mean ± SEM. Significance is indicated by *P ≤ 0.05, ***P ≤ 0.001, ****P ≤ 0.0001.

We next generated an independent *UBXD18*^*−*^ mutant to confirm that the UBXD18 protein regulates sensitivity to decanoic acid (Fig. 3*A* and *SI Appendix*, Fig. S4*A*). This recapitulated mutant was produced by insertion of a 1,592-bp blasticidin resistance sequence into the central region of the *ubxd18* gene, and was confirmed by loss of gene expression (*SI Appendix*, Fig. S4 *A* and *C*). UBXD18 activity was also reintroduced to *UBXD18*^*−*^ by expression of *ubxd18* complementary DNA (cDNA) in the mutant (*SI Appendix*, Fig. S4*B*), where the presence of green fluorescent protein (GFP)-UBXD18 was confirmed by Western blot analysis (*SI Appendix*, Fig. S4*D*). Wild-type, knockout (*UBXD18*^−^), and rescue (*UBXD18*^*−*/+^) cells were then assessed for the effect of decanoic acid on growth to quantify resistance (Fig. 3*B*), with IC_50_ values indicating comparative sensitivity (Fig. 3*C*). This analysis demonstrated that *UBXD18*^*−*^ was partially resistant to the effect of decanoic acid, with an IC_50_ value twofold higher than that of the parental cell line (*UBXD18*^*−*^ IC_50_ 37 µM, compared with wild-type IC_50_ 18 µM) (Fig. 3*C*). Reintroducing *ubxd18* gene expression restored a decanoic acid-sensitive phenotype (*UBXD18*^*−*/+^, IC_50_ 13 µM) (Fig. 3*C*). To establish if UBXD18 confers partial resistance to the other medium-chain fatty acids provided in the MCT diet, *UBXD18*^−^ was also assessed for the effect of octanoic acid on growth (*SI Appendix*, Fig. S5), where no significant change in sensitivity was observed (wild-type, IC_50_ 79 µM; *UBXD18*^−^, IC_50_ 87 µM), suggesting structural specificity in the targeting of UBXD18 by decanoic acid.

The role of UBXD18 in both regulating mTORC1 activity and controlling the effect of decanoic acid in reducing mTORC1 activity was investigated by Western blot analysis. Here, 4E-BP1 phosphorylation was monitored in wild-type, *UBXD18*^*−*^, and *UBXD18*^*−*/+^ in the presence of decanoic acid. Loss of UBXD18 caused a decrease in p-4E-BP1 levels compared with wild type, and p-4E-BP1 levels were restored by reintroduction of the protein (*UBXD18*^*−/+*^) (Fig. 3*D*). Analyzing p-4E-BP1 levels following decanoic acid treatment (60 µM for 1 or 24 h) showed that *UBXD18*^*−*^ was unresponsive to treatment (Fig. 3*E*), and *UBXD18*^*−*/+^ showed restored decanoic acid sensitivity following 24-h treatment (Fig. 3*F*).

### Characterization of UBXD18 as a Binding Partner for Ddp97.

**Fig. 4.**
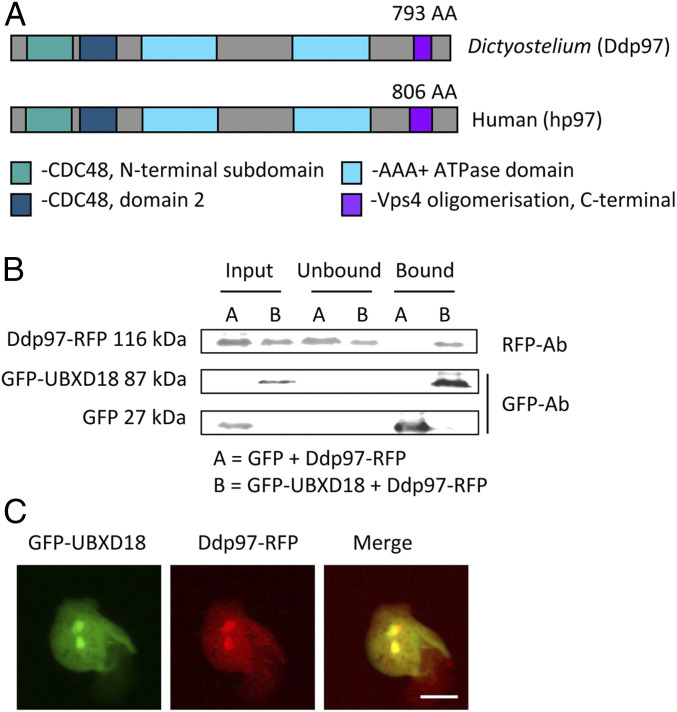
*Dictyostelium* UBXD18 interacts with Ddp97. (*A*) Schematic of the *Dictyostelium* (Ddp97) and human (hp97) p97 proteins, showing 78% sequence identity. (*B*) Cell lysates of *Dictyostelium* cells coexpressing GFP-UBXD18 (or free-GFP as a control) and Ddp97-RFP were subjected to immunoprecipitation with GFP trap beads and the interaction was analyzed by Western blot using anti-GFP and anti-RFP antibodies (representative of three independent experiments). Ddp97-RFP was pulled down by GFP-UBXD18 and not by the GFP-only control. (*C*) Colocalization of GFP-UBXD18 and Ddp97-RFP by visualizing fluorescence in live cells (representative of three independent experiments). (Scale bar, 10 µm.)

Our results demonstrate that UBXD18 controls sensitivity to decanoic acid in both growth and mTORC1 regulation in *Dictyostelium*. Since UBXD18 contains a p97 interaction motif (20, 49) (Fig. 2*B*) and the *Dictyostelium* p97 (Ddp97) and human p97 (hp97) proteins show conserved domain structure and a high sequence identity (78%) (Fig. 4*A*), we used immunoprecipitation analysis to investigate an interaction between UBXD18 and Ddp97. In these experiments, cell lines were generated coexpressing *Dictyostelium* GFP-UBXD18 (or free GFP as a control) and Ddp97-RFP (red fluorescent protein) (*SI Appendix*, Fig. S4). To monitor binding, cell lysates were mixed with agarose beads coated with anti-GFP antibody, and the interacting proteins were isolated and analyzed using Western blot with anti-GFP and anti-RFP antibodies. Ddp97-RFP was found to bind to GFP-UBXD18 but not to free GFP (Fig. 4*B*), suggesting that UBXD18 interacts with Ddp97. In support of this, GFP-UBXD18 and Ddp97-RFP were found to colocalize throughout the cytoplasm and nucleus (Fig. 4*C*), consistent with that suggested for the mammalian homologs (50, 51).

### Investigating the Effect of Decanoic Acid on Ddp97 Activity.

**Fig. 5.**
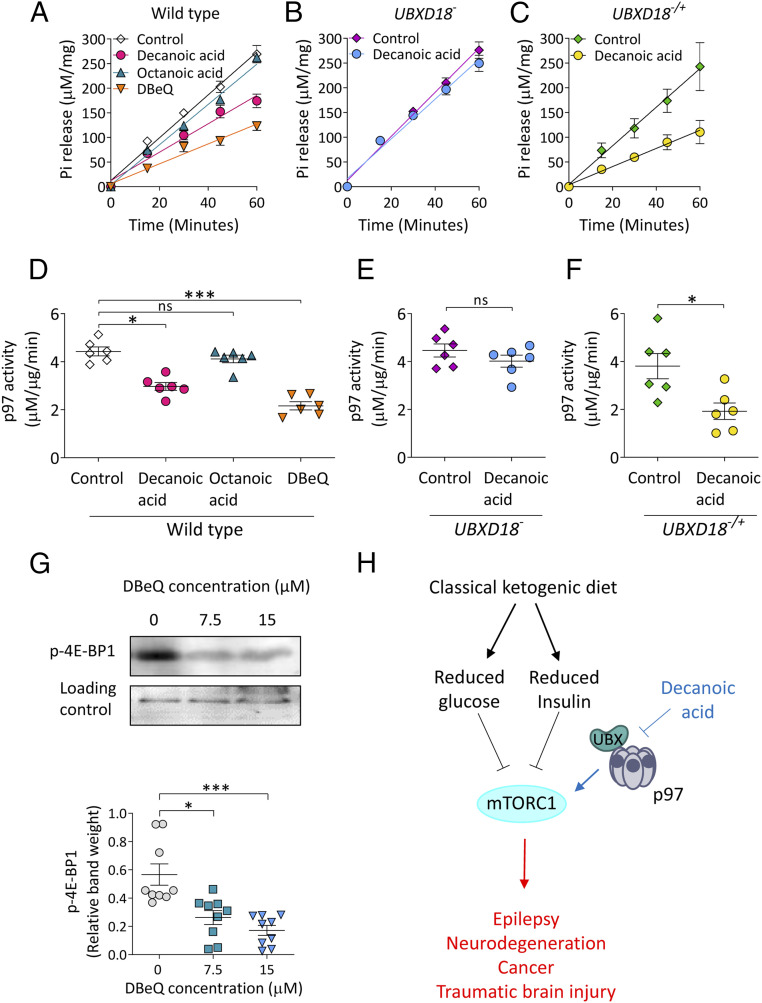
Decanoic acid inhibits Ddp97 activity in a UBXD18-dependent manner. (*A*) Analysis of phosphate (Pi) release from an ATP hydrolysis reaction with Ddp97-RFP isolated from wild-type *Dictyostelium* cells treated with decanoic acid (60 µM), octanoic acid (120 µM), or DBeQ (7.7 µM) for 24 h (*n* = 6). (*B* and *C*) Phosphate release was analyzed from ATP hydrolysis reactions with Ddp97-RFP isolated from (*B*) *UBXD18*^*−*^ and (*C*) *UBXD18*^*−/+*^ treated with decanoic acid (60 µM) for 24 h (*n* = 6). (*D*–*F*) Specific enzyme activity (phosphate [µM] released relative to protein concentration [µg]) was plotted versus time (min) and subjected to linear regression analysis. The gradient was plotted, representing the rate of ATP hydrolysis (Ddp97 activity µM phosphate⋅µg protein^−1^⋅min^−1^) for (*D*) wild type (Kruskal–Wallis test with Dunn’s post hoc test), (*E*) *UBXD18*^*−*^ (Mann–Whitney *t* test), and (*F*) *UBXD18*^*−/+*^ (Mann–Whitney *t* test) (*n* = 6). (*G*) Analysis of p-4E-BP1 levels in wild-type cells treated for 24 h with DMSO control (0 μM) or p97 inhibitor (DBeQ) at 7.5 or 15 µM (*n* = 9) (Kruskal–Wallis test with Dunn’s post hoc test). MCCC1 served as a loading control. (*H*) Simplified schematic portraying the canonical effect of the classical ketogenic diet on mTORC1 through reduced glucose and insulin (black), downstream diseases displaying elevated mTORC1 (red), and a role of decanoic acid through a UBXD18- and p97-mediated pathway (blue). Data represent the mean ± SEM. Significance is indicated by *P ≤ 0.05, **P ≤ 0.01, ***P ≤ 0.001; ns, not significant, *P* > 0.05.

An interaction between UBXD18 and Ddp97 suggests a role for UBXD18 in regulating Ddp97 function, and thus implicates Ddp97 in the cellular changes caused by decanoic acid in *Dictyostelium*. To analyze this, we assessed the effect of decanoic acid on Ddp97 activity by treating wild-type cells expressing Ddp97-RFP with decanoic acid, octanoic acid, or the established selective p97 inhibitor DBeQ (*N*2,*N*4-dibenzylquinazoline-2,4-diamine) (16, 52) for 24 h before immunoprecipitation of Ddp97-RFP and direct assessment of ATPase activity (53) (Fig. 5). Treatment with decanoic acid (60 µM) decreased Ddp97-RFP ATPase activity (Fig. 5 *A* and *D*), while octanoic acid (120 µM) had no significant effect, and the p97 inhibitor DBeQ (7.5 µM) also significantly reduced activity. Having demonstrated that decanoic acid but not octanoic acid acts to reduce Ddp97-RFP activity, we assessed the effect of decanoic acid on this activity in cells lacking UBXD18 (*UBXD18*^*−*^) (Fig. 5 *B* and *E*), where decanoic acid did not affect Ddp97-RFP activity. Reintroduction of UBXD18 into *UBXD18*^*−*/+^ also restored the decanoic acid-dependent reduction of Ddp97-RFP activity (Fig. 5 *C* and *F*). These findings suggest that decanoic acid functions through a mechanism involving UBXD18 to reduce Ddp97 activity in *Dictyostelium*.

Having established an effect of decanoic acid in inhibiting *Dictyostelium* p97 activity, we assessed the effect of inhibiting p97 on mTORC1 activity by using DBeQ. In these experiments, cells were treated with DBeQ (7.5 and 15 μM for 24 h), and cell lysates were analyzed for 4E-BP1 phosphorylation by Western blot (Fig. 5*G*). Treatment with DBeQ caused a significant reduction in p-4E-BP levels at both 7.5 and 15 μM (Fig. 5*G*). This was consistent with a comparable inhibition of growth caused by these concentrations of DBeQ (*SI Appendix*, Fig. S6). These findings suggest that the inhibitory effect of decanoic acid on *Dictyostelium* p97 activity may be sufficient to explain the observed mTORC1 and growth inhibition effects (Fig. 5*H*).

### Decanoic Acid Reduces mTORC1 Activity in Rat Hippocampal Slices.

**Fig. 6.**
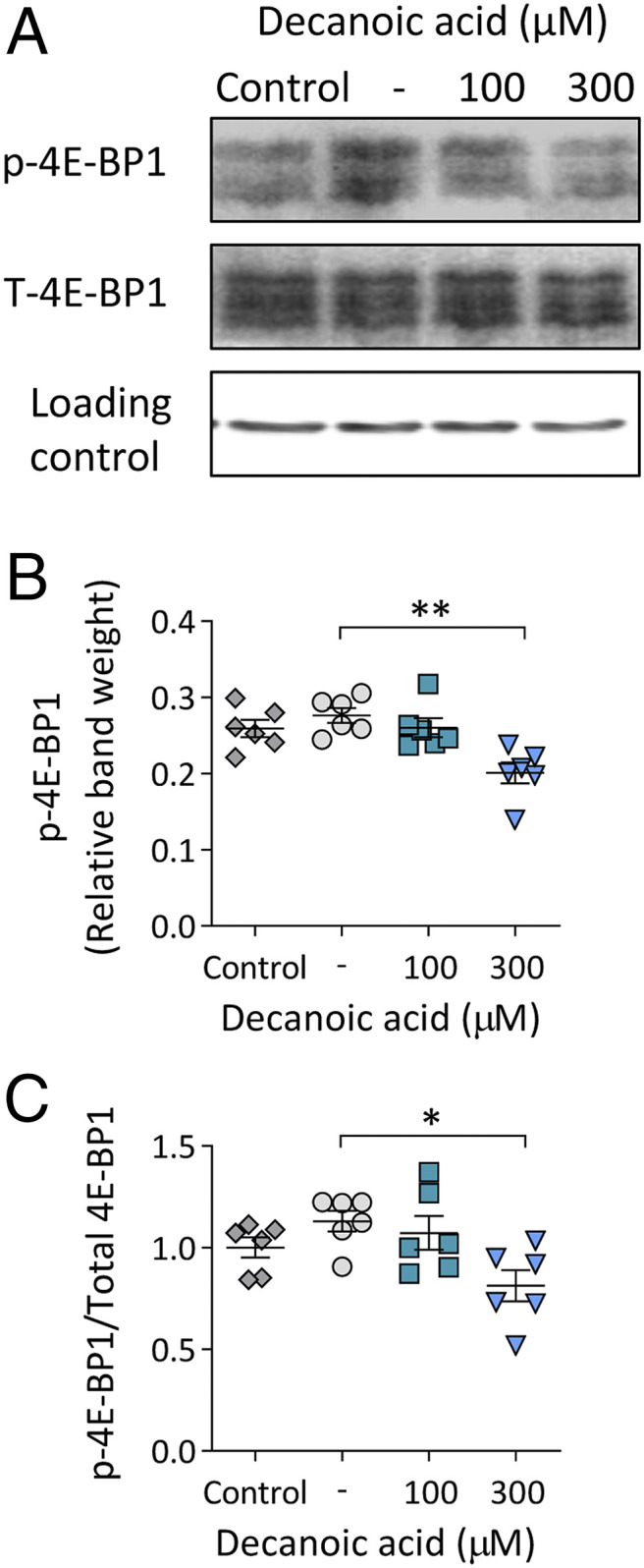
Decanoic acid causes a reduction in p-4E-BP1 levels in rat hippocampal brain slices. (*A*) Rat hippocampal brain slices in artificial cerebrospinal fluid (control), treated for 1 h with DMSO solvent control (-), 100 µM decanoic acid, or 300 µM decanoic acid were analyzed for p-4E-BP1 and total-4E-BP1 (T-4E-BP1), with beta-actin as a loading control. Multiple bands correspond to the three isoforms of 4E-BP1. (*B*) Relative band weights were plotted for p-4E-BP1 (*n* = 6) (Kruskal–Wallis test with Dunn’s post hoc test). (*C*) The ratio of p-4E-BP1/T-4E-BP1 was used as a readout for mTORC1 (*n* = 6) (Kruskal–Wallis test with Dunn’s post hoc test). Data represent the mean ± SEM. Significance is indicated by *P ≤ 0.05, **P ≤ 0.01.

Having identified an effect of decanoic acid in reducing mTORC1 activity independent of altered glucose levels and insulin signaling in *Dictyostelium*, we sought to translate these findings to a mammalian system. Here, we employed rat hippocampal slice preparations maintained in artificial cerebrospinal fluid, ensuring constant glucose levels in the absence of insulin, treated with decanoic acid (100 and 300 μM for 1 h) prior to assessing mTORC1 activity by Western blot analysis of p-4E-BP1 and total-4E-BP1 levels (Fig. 6). Decanoic acid at 100 μM had no significant effect on p-4E-BP1 levels (Fig. 6 *A* and *B*) but significantly decreased p-4E-BP1 levels at 300 μM (Fig. 6 *A* and *B*) and neither concentration altered total-4E-BP1 levels (Fig. 6*A* and *SI Appendix*, Fig. S7). These combined data showed a decrease in p-4E-BP1/total-4E-BP1 levels following treatment with 300 μM decanoic acid (Fig. 6*C*). Consistent with that observed in *Dictyostelium*, no change in p-AKT(Thr308)/total-AKT or p-AKT(Ser473)/total-AKT was observed in rat hippocampal slices following decanoic acid treatment (*SI Appendix*, Fig. S8).

### Decanoic Acid Reduces mTORC1 Activity in Patient-Derived Astrocytes.

**Fig. 7.**
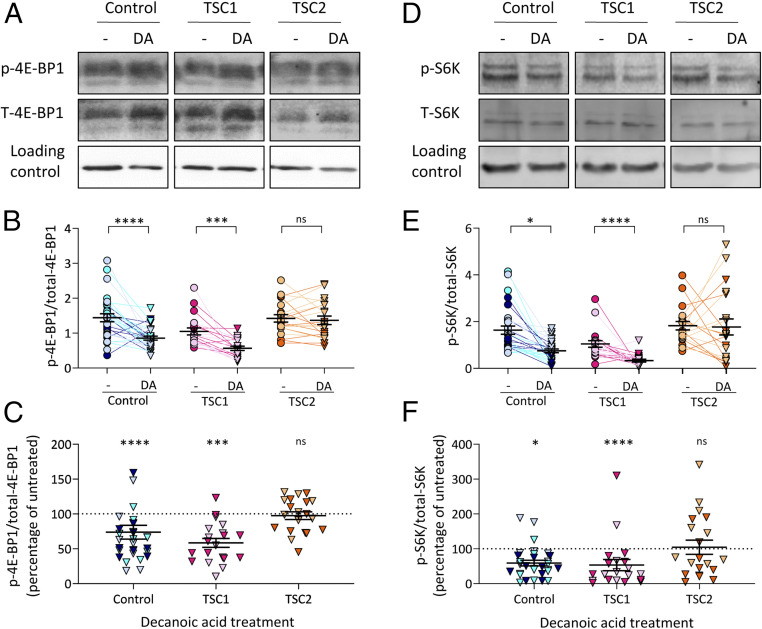
Decanoic acid decreases the ratio of p-4E-BP1/total-4E-BP1 and p-S6K/total-S6K in iPSC-derived astrocytes from healthy individuals and patients with TSC1 mutations. (*A*) Astrocytes derived from iPSCs from three healthy individuals (control), two patients with mutations in TSC1, or two patients with mutations in TSC2 were treated with DMSO solvent control (-) or 300 µM decanoic acid (DA) for 24 h before analysis of p-4E-BP1 and T-4E-BP1 levels, using beta-actin as a loading control. (*B*) The ratio of p-4E-BP1/total-4E-BP1 was used as a readout for mTORC1 (*n* = 10 per patient) (nested *t* test). (*C*) The percentage change in p-4E-BP1/total-4E-BP1 levels following decanoic acid treatment is plotted normalized to the untreated samples (DMSO solvent control) (*n* = 10 per patient) (nested *t* test). (*D*) The same samples were analyzed for levels of p-S6K and total-S6K, using beta-actin as a loading control. (*E*) The ratio of p-S6K/total-S6K was used as a readout for mTORC1 (*n* = 10 per patient) (nested *t* test). (*F*) The percentage change in p-S6K/total-S6K levels following decanoic acid treatment is plotted normalized to the untreated samples (DMSO solvent control) (*n* = 10 per patient) (nested *t* test). Data from individual patients are differentiated by color. Data represent the mean ± SEM. Significance is indicated by *P ≤ 0.05, ***P ≤ 0.001, ****P ≤ 0.0001; ns, *P* > 0.05.

To investigate a potential role for decanoic acid in reducing mTORC1 activity in a clinically relevant setting, we monitored this activity in astrocytes derived from patients with tuberous sclerosis complex. mTORC1 signaling is reportedly activated in astrocytes during epileptogenesis (54), with this cell type believed to play a significant role in seizure induction (55–57). In these experiments, induced pluripotent stem cells (iPSCs) derived from patients with mutations in genes encoding either TSC1 or TSC2, or healthy controls, were differentiated into astrocytes (58) and treated with decanoic acid (300 μM for 24 h) prior to Western blot analysis of p-4E-BP1 and total-4E-BP1 levels. Decanoic acid treatment caused a reduction in the ratio of p-4E-BP1/total-4E-BP1 in astrocytes derived from healthy (control) patients (Fig. 7 *A*–*C*), confirming a role for decanoic acid in regulating mTORC1 activity in human cells in the absence of altered glucose or insulin signaling. Decanoic acid also caused a reduction in the ratio of p-4E-BP1/total-4E-BP1 in astrocytes derived from patients with mutations in TSC1 (Fig. 7 *A*–*C*). However, decanoic acid had no significant effect on this ratio in astrocytes derived from patients with mutations in TSC2 (Fig. 7 *A*–*C*). In these assays, levels of p-4E-BP1 were not significantly altered following treatment (*SI Appendix*, Fig. S9 *A* and *B*), but the reduction in the ratio of p-4E-BP1/total-4E-BP1 was caused by a significant increase in levels of total-4E-BP1 (*SI Appendix*, Fig. S9 *C* and *D*). Interestingly, variability was observed between cells from different TSC2 patients, with cells from one patient showing no significant change in total-4E-BP1 levels following decanoic acid treatment, while cells from another patient showed a significant increase (*SI Appendix*, Fig. S10 *A* and *B*).

To confirm the effect of decanoic acid in reducing mTORC1 activity in patient-derived astrocytes, we also analyzed p70 S6 kinase (S6K) phosphorylation levels. For this activity, decreased phosphorylation at threonine 389 (p-S6K) indicated decreased mTORC1 activation (59) (Fig. 7*D*). Analysis of the same protein extracts showed that decanoic acid treatment also caused a reduction in the ratio of p-S6K/total-S6K in astrocytes derived from control patients and patients with mutations in TSC1 (Fig. 7 *D*–*F*) but showed no effect in astrocytes derived from patients with mutations in TSC2 (Fig. 7 *D*–*F*). The reduction in this ratio was caused by a significant reduction in levels of p-S6K (*SI Appendix*, Fig. S9 *E* and *F*) without changes in total-S6K levels (*SI Appendix*, Fig. S9 *G* and *H*). Variability was also observed between cells from individual TSC2 patients, with cells from one patient showing no significant change in p-S6K levels following decanoic acid treatment, while cells from the other showed a significant decrease (*SI Appendix*, Fig. S10 *C* and *D*).

## Discussion

Ketogenic diets are well-established treatments for epilepsies (21) and cancers (6) with exciting potential for use in other areas of health such as treating neurodegenerative disorders (7); however, the therapeutic mechanisms underlying these effects are poorly understood. In this study, we have used *Dictyostelium* as a tractable model to establish that decanoic acid, one of the major constituents of the MCT ketogenic diet, inhibits mTORC1 signaling. In order to identify the mechanism behind this effect, we showed that the effects of decanoic acid on mTORC1 are dependent on a UBX domain-containing protein (UBXD18). We demonstrated that decanoic acid inhibits the activity of the ubiquitous binding partner of UBXD18, p97, in a UBXD18-dependent manner, and further showed that direct p97 inhibition is sufficient to reduce mTORC1 signaling in *Dictyostelium*. We translated this effect of decanoic acid into a rat hippocampal slice model, demonstrating decanoic acid-dependent inhibition of mTORC1 signaling in the absence of glucose deprivation or insulin signaling. Finally, we employed astrocytes derived from patients with mutations in TSC1 or TSC2 associated with dysregulated mTORC1 activity (11, 58) to demonstrate that treatment with decanoic acid results in decreased mTORC1 activation in healthy patients and patients with TSC1 mutations.

We provide mechanistic insight for the role of decanoic acid in regulating mTORC1 activity in *Dictyostelium*, through a UBX domain-containing protein and *Dictyostelium* p97. UBX proteins have previously been shown to directly regulate the activity of p97 in human cells (20). By employing a specific inhibitor for p97 (DBeQ), we also confirmed that pharmacological inhibition of *Dictyostelium* p97 decreased mTORC1 activity and cell proliferation (16) (Fig. 5*G* and *SI Appendix*, Fig. S6), suggesting that decanoic acid-induced p97 inhibition could explain the inhibitory effects on mTORC1 activity and growth in the model system (Fig. 5*H*). Although the role of human p97 in regulating mTORC1 has yet to be established, there is evidence that inhibition of p97 results in the disruption of amino acid homeostasis, potentially leading to the observed reduction in mTORC1 (16, 52). Our data provide an indication that decanoic acid may prevent the interaction of *Dictyostelium* p97 with its regulatory cofactor UBXD18, thus reducing *Dictyostelium* p97 activity and leading to a reduction in mTORC1. Interactions between long-chain fatty acids and UBX domain-containing proteins have previously been implicated in blocking p97 activity in mammalian cells (60), suggesting that regulation of p97 via an interaction of decanoic acid with a UBX domain-containing protein represents a viable potential mechanism in humans.

Inhibition of p97 has been proposed as a treatment for cancer and epilepsy (61, 62). In cancers, expression of p97 is increased to manage excessive proteotoxic stress (63), and thus p97 inhibitors are in clinical trials as cancer treatments (64, 65). p97 also plays a role in a common form of epilepsy (autosomal dominant juvenile myoclonic epilepsy), where a mutation in a GABA_A_ receptor subunit (A322D mutation in the α1-subunit) results in misfolding and rapid ERAD of the α1-subunit, resulting in epilepsy (62, 66). By inhibiting p97, α1(A322D) subunits have more time to fold in the endoplasmic reticulum, allowing functional GABA_A_ receptors to form and act as inhibitory ion channels (62). Our findings suggest that further analysis of a role for decanoic acid in inhibiting p97 and mTORC1 may identify associated therapeutic benefits.

Our data suggest a mechanism for decanoic acid in regulating mTORC1 independent of glucose and insulin. The classical ketogenic diet is known to reduce mTORC1 activation, with a proposed mechanism through lowering glucose and insulin levels (1–4) associated with the therapeutic effects of the classical ketogenic diet in treating epilepsy. However, the role of the MCT ketogenic diet fatty acids in this function has not previously been reported. Several studies have suggested that decanoic acid in the MCT diet provides therapeutic benefits in seizure control (5, 26, 27, 67), and here we have identified that in *Dictyostelium*, decanoic acid inhibits mTORC1 activity under conditions of constant glucose and in the absence of insulin (Fig. 1*E*). Since we have translated this effect to both a rat hippocampal slice model (Fig. 6) and patient-derived astrocytes (Fig. 7), again under conditions of constant glucose and in the absence of insulin signaling, our data support an evolutionarily conserved effect of decanoic acid, provided in the MCT diet, distinct from that proposed for the classical ketogenic diet.

The mTORC1 pathway acts as a regulator for cell growth through the phosphorylation of targets activating anabolic processes and inhibiting catabolic processes. In this role, mTORC1 inhibition has been credited with providing therapeutic effects in many areas of health, including treating epilepsy and neurodegenerative disorders, preventing the proliferation of cancer cells, improving cognitive function after traumatic brain injury, and increasing longevity (8–10). mTORC1 inhibition has also been investigated as a therapeutic target for the neurodevelopmental disorder TSC associated with deregulated mTORC1 activity (11, 13, 14, 68). The cause of epilepsy in TSC patients is often related to the formation of cortical tubers, characterized by cortical dyslamination, dysplastic neurons, and astrogliosis (69). Although astrogliosis may be secondary to neuronal pathology, studies in animals and humans suggest that TSC involves a primary defect in astrocytes (55), supported by mTOR dysregulation (70). In cell culture, however, patient-derived cells have been suggested to employ a feedback loop to regulate the TSC and maintain balanced mTOR signaling (71). Importantly, we show that decanoic acid reduces mTORC1 signaling in cells derived from both healthy individuals and patients with TSC mutations, suggesting that decanoic acid functions independent of disease state and shows efficacy in cells derived from patients with disease-associated mutations (Fig. 7). However, heterogeneity between astrocytes derived from patients with mutations in TSC2 were observed (*SI Appendix*, Fig. S10), which suggests that decanoic acid might provide differential efficacy between individuals.

Inhibition of mTORC1 has been suggested to provide wide-ranging clinical benefits, including as a treatment for cancer, epilepsy, neurodegenerative diseases, and aging (14, 72–76). The recent development of new pharmacological inhibitors for mTORC1 inhibition, based around rapamycin, have focused on identifying “rapalogues” without the toxicity associated with rapamycin (68). Alternately, inhibition of this pathway is possible through dietary treatment, namely the classical ketogenic diet (1–4); however, this diet is highly restrictive for patients, resulting in low compliance. Our data suggest that a nonketogenic decanoic acid-rich diet that is less restrictive and avoids the potential adverse effects of ketosis could also provide therapeutic effects through inhibiting this pathway. Thus, further investigation into dietary decanoic acid may provide a useful approach to treat mTORopathies in addition to providing a range of associated health effects.

## Materials and Methods

Detailed methods are available in *SI Appendix*.

### *Dictyostelium* Growth Assay.

*Dictyostelium* cells in the presence of compound or a dimethyl sulfoxide (DMSO) control were grown at 22 °C in HL5, and the cell number was quantified.

### Cell Development Assays.

Exponentially growing cells were developed on nitrocellulose filters on absorbent pads soaked in phosphate buffer containing compound or DMSO control. Developmental phenotypes were imaged after 24 h.

### *Dictyostelium* Mutant Library Screen.

A *Dictyostelium* mutant library was grown alongside wild-type cells in decanoic acid or DMSO control and screened for the appearance of resistant colonies. Resistant mutants were isolated and selected isogenically and mutation sites were identified using inverse PCR.

### Knockout Generation.

*UBXD18*^*−*^ cells were generated by homologous integration. A knockout cassette was generated by PCR amplification over the REMI insertion site using genomic DNA extracted from the mutant. The PCR product was electroporated into *Dictyostelium* wild-type cells.

### Creation of GFP-UBXD18 and Ddp97-RFP Constructs.

*ubxd18* or *Dictyostelium p97* cDNA was cloned into extrachromosomal vectors before electroporation into *Dictyostelium* cells.

### Western Blotting.

Samples were prepared in Laemmli buffer, separated by sodium dodecyl sulfate polyacrylamide gel electrophoresis, transferred to membranes, and probed with antibodies as described. Blots were visualized using the Odyssey CLx Imager (LI-COR), quantified using LI-COR Image Studio, and normalized to the loading control or total (unphosphorylated) protein (as specified).

### Immunoprecipitation.

GFP-TrapA beads were used for immunoprecipitation of GFP-fusion proteins before analysis by Western blot.

### ATPase Assay.

*Dictyostelium* cells, expressing Ddp97-RFP, were treated for 24 h with compound. RFP-TrapA beads were used for the immunoprecipitation of RFP-tagged proteins and ATPase activity was monitored as described (53).

### Rat Hippocampal Slice Preparation for Western Blot.

Adult Sprague–Dawley rats were used to prepare hippocampal slices as detailed (30). Animal experiments were conducted in accordance with Animals (Scientific Procedures) Act 1986 and approved by the local University College London ethics committee.

### Patient-Derived Astrocyte Differentiation and Preparation for Western Blot.

iPSCs from three individual control patients, two patients with TSC1 mutations, and two patients with TSC2 mutations were differentiated into astrocytes as described (58). The following cell lines were obtained from the National Institute of General Medical Sciences Human Genetic Cell Repository at the Coriell Institute for Medical Research: GM23964, GM23973, GM06149, GM02332, GM03958, and GM06102. An additional control line was kindly provided by Eleonora Aronica, Amsterdam Universitair Medisched Centra. All experiments were exempt from approval of the Medical Ethical Toetsingscommissie, the institutional review board of the Vrije Universiteit Medical Center.

## Supplementary Material

Supplementary File

## Data Availability

All data are available in the text and *SI Appendix*.
