# Supplementary material for: Decanoic acid inhibits mTORC1 activity independent of glucose and insulin signaling
Source: Proc Natl Acad Sci U S A. 2020 Sep 2;117(38):23617–25. doi: 10.1073/pnas.2008980117 (PMC7519326; doi:10.1073/pnas.2008980117)
Supplement: Supplementary File [file pnas.2008980117.sapp.pdf]

Supplementary figures

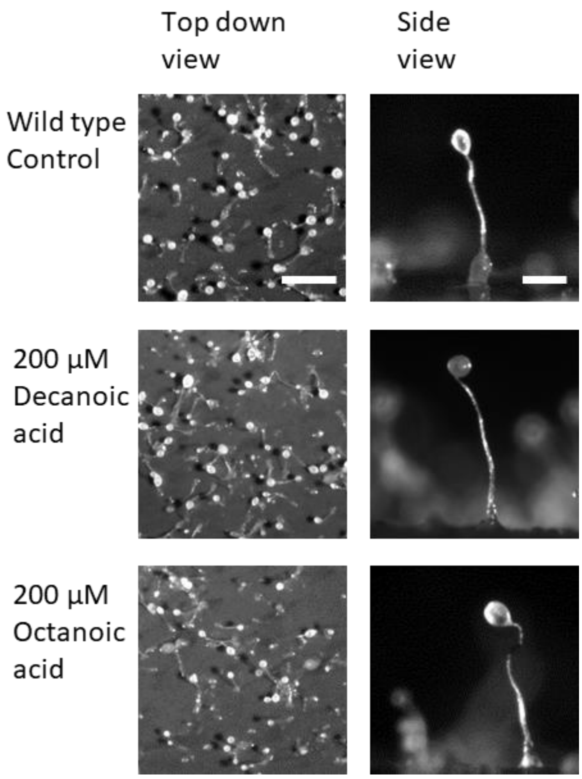

S1. Medium chain fatty acids do not inhibit *Dictyostelium* development. Qualitative evaluation of developmental effects of medium chain fatty acids on *Dictyostelium*. Phenotypes were monitored following starvation of wild type *Dictyostelium* cells for 24 hours on nitrocellulose filters in the presence of compound or solvent (DMSO) control. Top down (scale bar 1 mm) and side on (scale bar 0.2 mm) images are shown as representative images of results from triplicate experiments.

www.pnas.org/cgi/doi/10.1073/pnas.2008980117

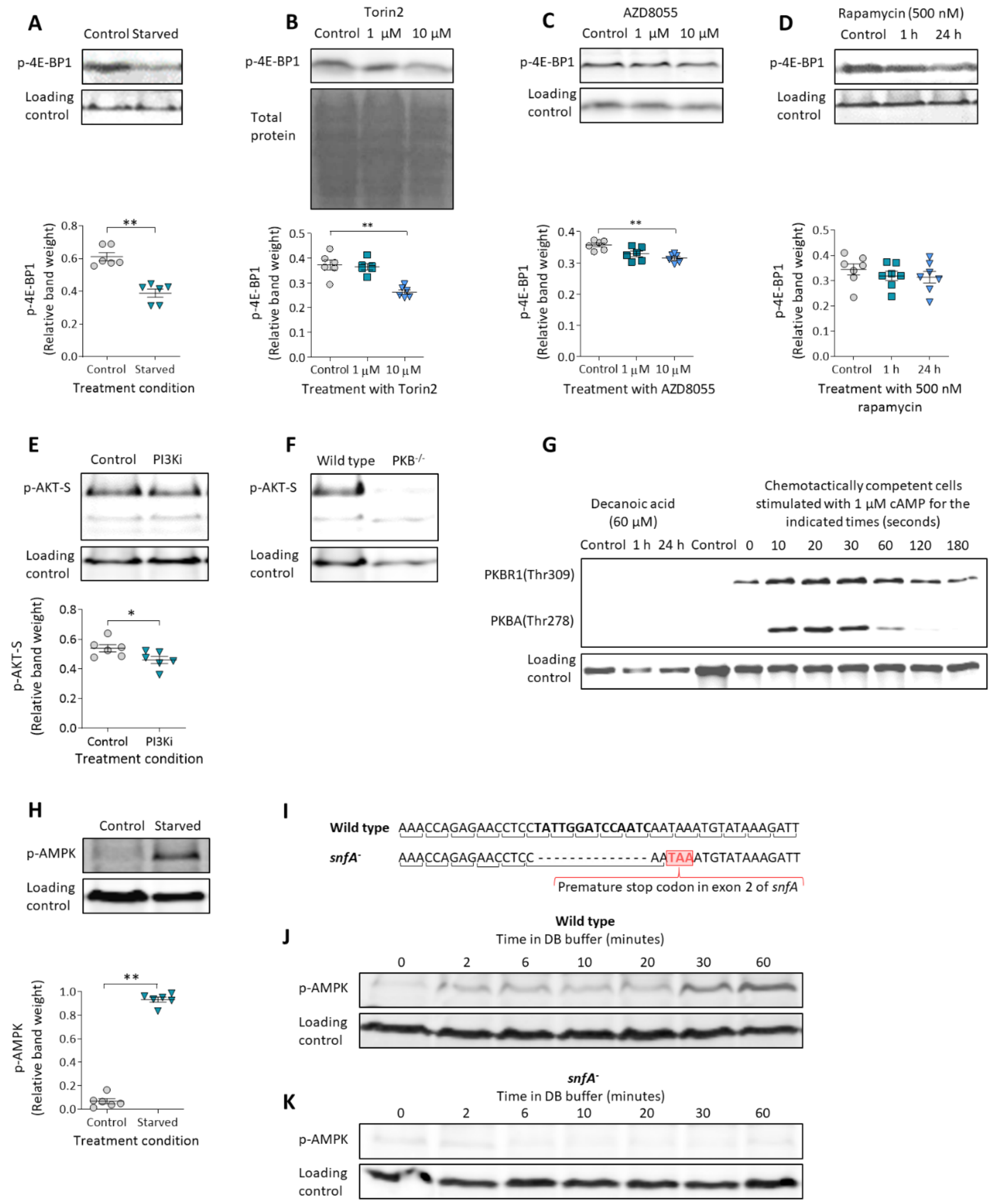

S2. Verification of p-4E-BP1, p-AKT-S and p-AMPK antibodies in *Dictyostelium*. Since no *Dictyostelium* 4E-BP1 null mutant was available, we validated the p-4E-BP1 antibody using controls of A) starvation and B) and C) mTOR inhibitors. A) Analysis of p-4E-BP1 levels in wild type cells untreated or starved for 10 minutes in nutrient free buffer (KK2), with methylcrotonyl-CoA carboxylase (MCCC1) as a loading control (n = 6) (Mann-Whitney t-test). B) Analysis of p-4E-BP1 levels in wild type cells untreated or treated with 1 or 10 µM Torin2 for 24 hours (n = 6), normalised to total protein (due to effects of Torin2 on mitochondria (1)) (Kruskal-Wallis test with Dunn's post hoc test). C) Analysis of p-4E-BP1 levels in wild type cells untreated or treated with 1 or 10 µM AZD8055 for 24 hours (n = 6), with MCCC1 as a loading

control (Kruskal-Wallis test with Dunn's post hoc test). D) Analysis of p-4E-BP1 levels in wild type cells untreated or treated with 500 nM rapamycin for 1 hour (h) or 24 hours (n = 7), with MCCC1 as a loading control (Kruskal-Wallis test with Dunn's post hoc test). E) AKT activity was evaluated in wild type cells treated with 60 µM PI3k inhibitor for 24 hours (LY294002), with MCCC1 as a loading control (n = 6) (Mann-Whitney t-test). F) A pkbA$^-$/pkgB$^-$ double knockout shows negligible AKT-substrate phosphorylation, with MCCC1 as a loading control. G) Chemoattractant-induced phosphorylation of T309 of PKBR1 and T278 of PKBA conserved activation loop, using the anti-phospho PKC (pan) antibody is unsuitable for monitoring p-AKT in growing cells treated with decanoic acid since this phosphorylation requires induction by chemoattractant (cAMP). MCCC1 served as a loading control. H) Analysis of p-AMPK levels in wild type cells, untreated or starved for 2 hours in nutrient free Development Buffer (DB), with MCCC1 as a loading control (n = 6) (Mann-Whitney t-test). I) The snfA mutant (snfA$^-$) was generated using the CRISPR/Cas9 technology according to Sekine et. al (2018) (2). This mutant has a frameshift mutation (deletion of 14 bp) in exon 2 of snfA, which leads to a premature stop in translation and therefore to a truncated protein. Starvation in DB buffer induces phosphorylation of AMPK in J) wild type, but not in K) snfA$^-$, with MCCC1 as a loading control (representative of 3 independent experiments). Data represent the mean ± SEM. Significance is indicated by * $p \leq 0.05$, ** $p \leq 0.01$, *** $P \leq 0.001$.

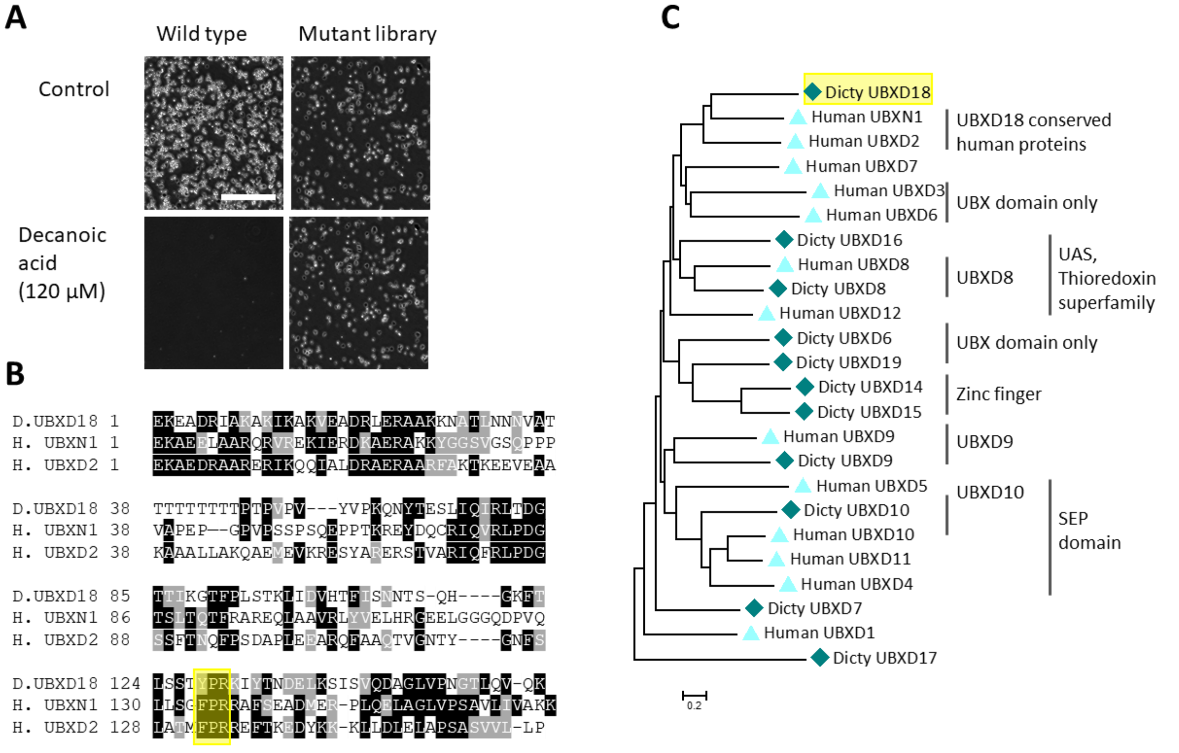

S3. The *Dictyostelium* UBXD18 protein, identified from a mutant library screen, contains the evolutionarily conserved UBX domain. A genetic screen of an insertional mutant library was carried out to identify mutants partially resistant to the growth inhibitory effects of decanoic acid. A) Representative images of a mutant library screen in decanoic acid are shown (scale bar 0.5 mm). The genetic screen identified a gene encoding the UBX domain containing protein 18 (UBXD18, DDB_G0276057) as being a potential target for decanoic acid. B) The two most highly conserved human proteins UBXN1 and UBXD2 share a common UBX domain (displayed as a multiple sequence alignment) with a highly conserved s3/s4 loop involved in binding p97 (highlighted). C) Cladistic analysis of *Dictyostelium* and human UBX domain contacting proteins confirm that UBXD18 is most conserved with human UBXN1 and UBXD2. Protein sequences were aligned using Clustal and the phylogenetic tree was generated using the neighbour joining method based on the alignment (MEGA).

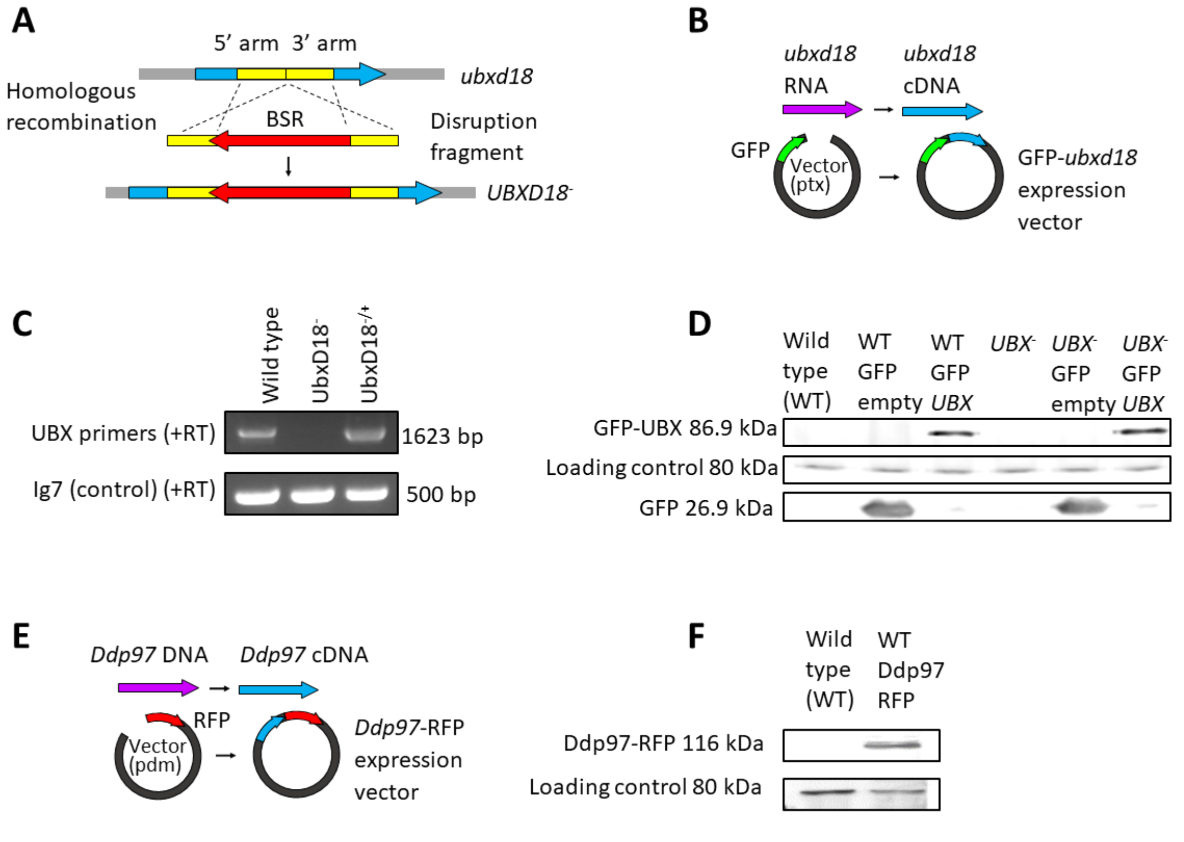

S4. Generation of *UBXD18*$^{-}$, *UBXD18*$^{-/+}$, and Ddp97-RFP cell lines A) Schematic for the generation of the *UBXD18*$^{-}$ cell line. (B) Schematic for the generation of a GFP-*ubxd18* expression vector. C) The absence of UBXD18 in the null cell line, and the presence of UBXD18 in the rescue cell line was confirmed by RT-PCR. D) The presence of GFP-UBXD18 (GFP-UBX) within the rescue cell line was confirmed by western blot analysis using an anti GFP antibody, with methylcrotonyl-CoA carboxylase (MCCC1) as loading control. E) Schematic for the generation of a *Ddp97*-RFP expression vector. F) The presence of Ddp97-RFP within the Ddp97-RFP expressing cell line was confirmed by western blot analysis using an anti RFP antibody with MCCC1 as a loading control.

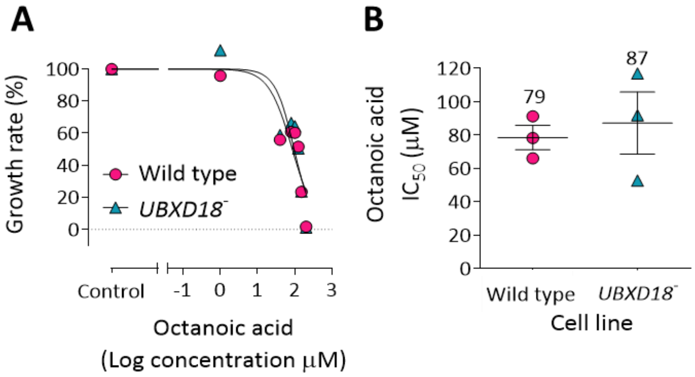

S5. UBXD18 does not regulate the sensitivity of *Dictyostelium* to octanoic acid. A) Growth analyses of wild type and *UBXD18*- *Dictyostelium* cells in octanoic acid are displayed as dose response curves of normalised cell density plotted against Log concentration (n = 3). B) IC_{50} values compared between wild type and *UBXD18*- cell lines (n = 3) (Mann-Whitney t-test). Data represent the mean ± SEM.

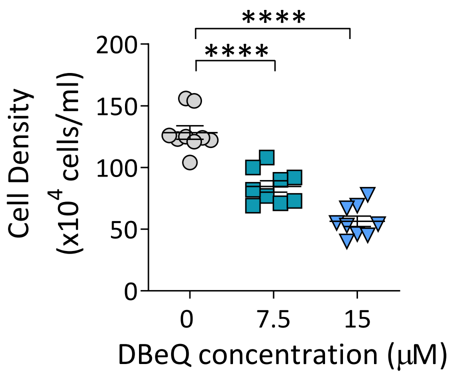

S6. p97 inhibitor DbeQ inhibits *Dictyostelium* growth. *Dictyostelium* cells (30 x 10^4 cells/ml) were grown for 24 hours in 0 μM, 7.5 μM, and 15 μM DbeQ, before cell growth was quantified (n = 9) (1-way ANOVA with Dunnett's post hoc test). Data represent the mean ± SEM. Significance is indicated by **** $p \le 0.0001$.

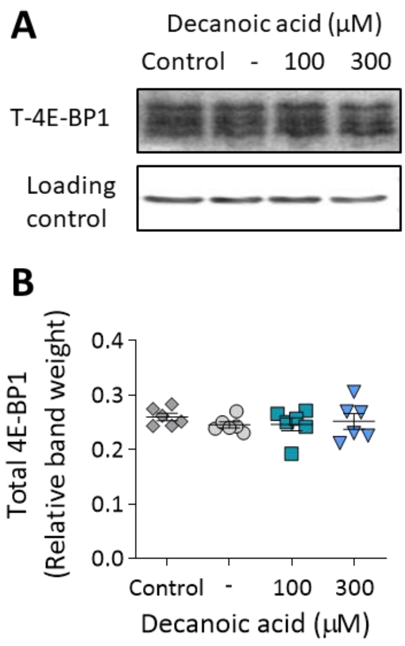

S7. Decanoic acid does not alter total-4E-BP1 levels in rat hippocampal brain slices. A) Rat hippocampal brain slices treated for 1 hour with aCSF (control), DMSO solvent control (-), 100 μM decanoic acid (DA) or 300 μM decanoic acid (DA) were analysed for total 4E-BP1 (T-4E-BP1), with beta-actin as a loading control. B) Relative band weights were plotted for total 4E-BP1 (n = 6) (Kruskal-Wallis test with Dunn’s post hoc test). Data represent the mean ± SEM.

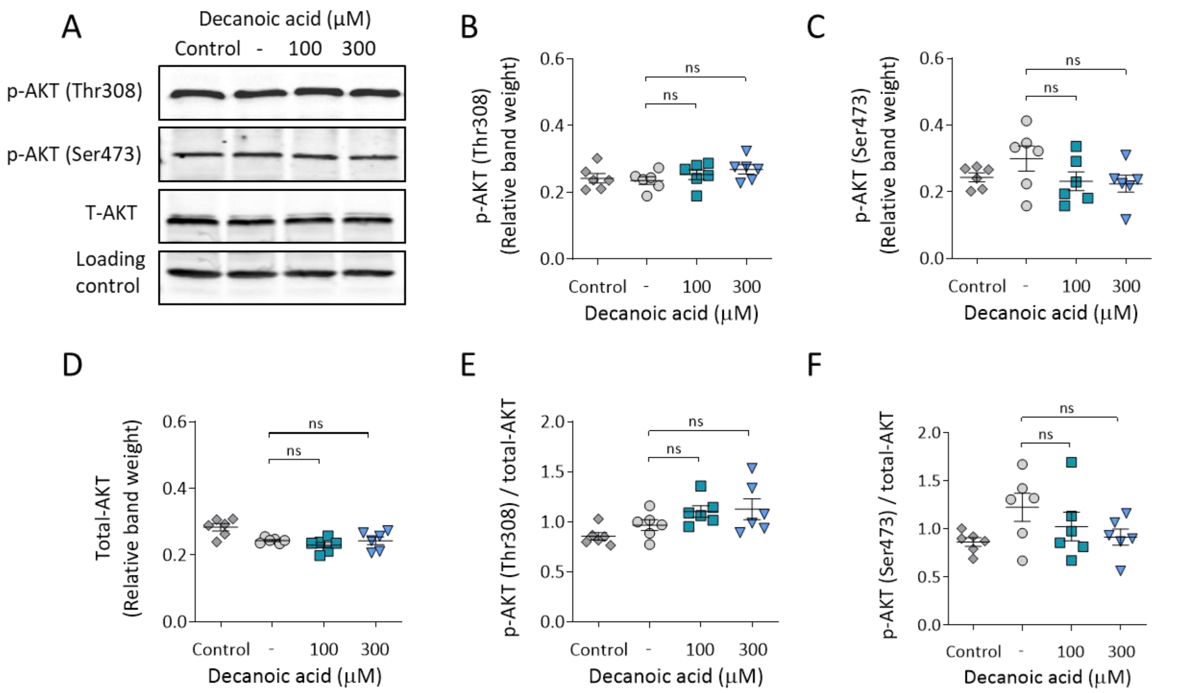

S8. Decanoic acid does not alter p-AKT in levels in rat hippocampal brain slices A) Rat hippocampal brain slices treated for 1 hour with artificial cerebral spinal fluid (aCSF) (control), DMSO solvent control (-), 100 µM decanoic acid (DA) or 300 µM decanoic acid (DA) were analysed for phosphorylation at Thr308 (phosphorylation controlled by the PI3K pathway) and Ser473 (required for full activation) and for total-AKT (T-AKT) levels, with beta-actin as a loading control. Relative band weights were plotted for B) phospho-AKT at Thr308 (n = 6) (Kruskal-Wallis test with Dunn's post hoc test) C) phospho-AKT at Ser473 (n = 6) (Kruskal-Wallis test with Dunn's post hoc test) and D) total-AKT (n = 6) (Kruskal-Wallis test with Dunn's post hoc test). The ratio of E) p-AKT(Thr308)/total-AKT (n = 6) (Kruskal-Wallis test with Dunn's post hoc test) and F) p-AKT(Ser473)/total-AKT (n = 6) (Kruskal-Wallis test with Dunn's post hoc test) was plotted. Data represent the mean ± SEM.

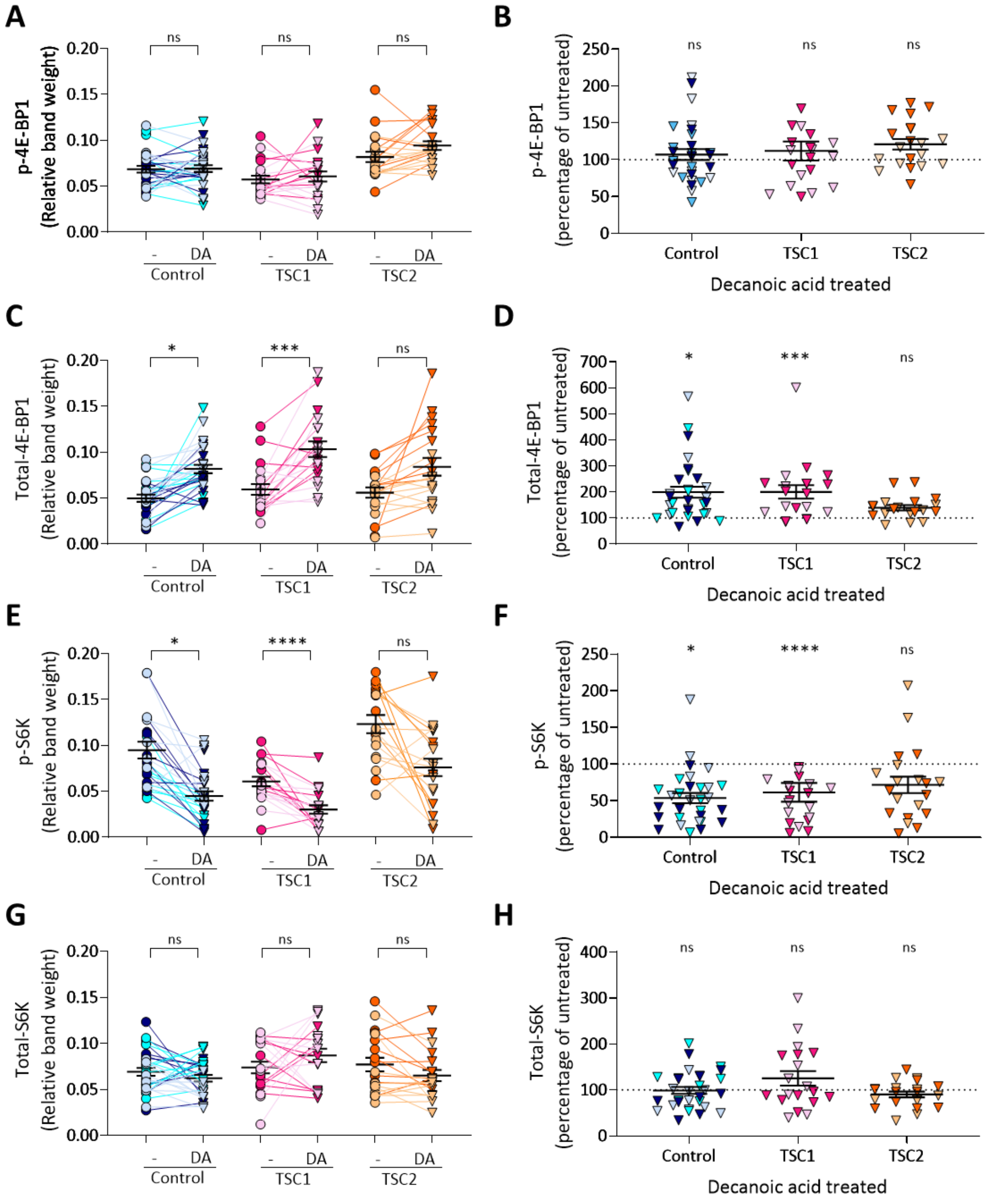

S9. Decanoic acid causes an increase in total-4E-BP1, and a decrease in p-S6K levels in astrocytes derived from patient iPSCs. Data used to evaluate the ratio of p-4E-BP1/total-4E-BP1 and p-S6K/total-S6K in figure 7. Astrocytes derived from iPSCs from three healthy patients (control), two patients with mutations TSC1 or two patients with mutations in TSC2 were treated with DMSO solvent control (-), or 300 µM decanoic acid (DA) for 24 hours before analysis of A) p-4E-BP1 (n = 10 per patient) (nested t-test), B) the percentage change in p-4E-BP1 following treatment (n = 10 per patient) (nested t-test), C) total 4E-BP1 levels (n = 10 per patient) (nested t-test), D) the percentage change in total-4E-BP1 following treatment (n = 10 per patient) (nested t-test), E) p-S6K (n = 10 per patient) (nested t-test), F) the percentage change in p-S6K following treatment (n = 10 per patient) (nested t-test), G) total-S6K (n = 10 per patient) (nested t-test) and H) the percentage change in total-S6K following treatment (n = 10 per patient) (nested t-test). Data from individual patients are differentiated by colour. Data represent the mean ± SEM. Significance is indicated by * $p \leq 0.05$, *** $p \leq 0.001$, **** $p \leq 0.0001$, ns $p > 0.05$.

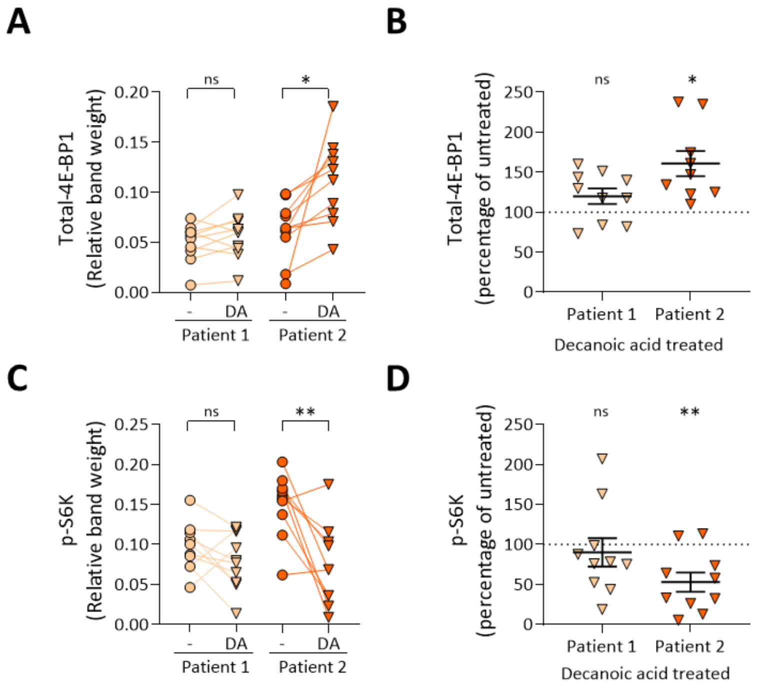

S10. Variation in TSC2 patient derived astrocyte response to decanoic acid. Astrocytes derived from iPSCs from two patients with mutations in TSC2 (Patient 1, hVS-417, and patient 2 hVS-424) were treated with DMSO solvent control (-), or 300 µM decanoic acid (DA) for 24 hours before analysis of A) total-4E-BP1 levels (n = 10) (Repeated measures ANOVA, with Sidak's multiple comparisons test), B) the percentage change in total-4E-BP1 levels following treatment (n = 10) (Repeated measures ANOVA, with Sidak's multiple comparisons test), C) p-S6K levels (n = 10) (Repeated measures ANOVA, with Sidak's multiple comparisons test), and D) the percentage change in p-S6K levels following treatment (n = 10) (Repeated measures ANOVA, with Sidak's multiple comparisons test). Data represent the mean ± SEM. Significance is indicated by * $p \leq 0.05$, ** $p \leq 0.01$, ns $p > 0.05$.

Supplementary methods

***Dictyostelium* growth assay.** *Dictyostelium* cells ($2x10^4$ cells/ml) in the presence of compound, decanoic acid, or octanoic acid (Alfa Aesar, A14788, A11149) or 0.2% DMSO control (Sigma, 276855) were grown at 22°C in HL5 (Formedium, HLB0103), and the cell number was quantified at 72 hours and then every 24 hours for 5 days.

***Dictyostelium* development assays.** Exponentially growing cells ($1x10^7$) were transferred to nitrocellulose filters (Millipore, HABP04700). Each filter was placed on an absorbent pad (Millipore, AP1004700), soaked in phosphate buffer, containing compound or DMSO control. Developmental phenotypes were imaged after 24 hours at 22°C.

***Dictyostelium* mutant library screen.** A *Dictyostelium* mutant library containing 28,000 insertional mutants (created by restriction enzyme mediated integration (REMI)) was screened alongside wild type (Ax4) cells for resistance to decanoic acid (120 µM). Cells (1.25 x 10^4 cells/ml) were grown in decanoic acid or solvent (DMSO) control (0.5%) in media containing 10 µg/ml blasticidin (Apollo Scientific, BIB4432) and screened for the appearance of resistant colonies. Resistant mutants were isolated, selected isogenically, and mutation sites identified using inverse PCR. Genomic DNA was extracted (Qiagen, 69504), digested (Rsa1, Thermo Fisher Scientific, ER1121) and circularised by ligation (T4 DNA ligase, Thermo Fisher Scientific, EL0014), and the genomic sequence flanking the mutagenic insertion site was amplified by PCR and sequenced.

Analysis of protein sequences Sequences of proteins were taken from dictybase (http://dictybase.org) or uniprot (http://www.uniprot.org). Regions of similarity between proteins were found using the Basic Local Alignment Search Tool (BLAST) (https://blast.ncbi.nlm.nih.gov). Sequences were aligned using Clustal (http://www.ebi .ac.uk/Tools/msa/clustalo/) which annotates sequence homology results across the whole protein. The program MEGA 7 was used to generate neighbour joining phylogenetic trees from these alignments (http://www.megasoftware.net). Domains were identified based on annotations from InterPro (https://www.ebi.ac.uk/interpro/).

Knockout generation. *UBXD18*$^-$ cells were generated by homologous integration. A knockout cassette was generated by PCR amplification over the REMI insert site using gDNA extracted from the mutant. This generated a construct with ~500 bp of gene homology either side of the REMI insert sequence conferring blasticidin resistance. The PCR product was spin column purified (Qiagen, 28104), then electroporated into *Dictyostelium* wild type (Ax2) cells with transformants selected with 10 µg/ml blasticidin.

Creation of GFP-UBXD18 and Ddp97-RFP constructs *ubxd18* or *Dictyostelium p97* (*Ddp97*) cDNA was cloned into extrachromosomal vectors (ptx-GFP, and pdm451 respectively), and the resulting plasmids were sequenced, before electroporation into *Dictyostelium* wild type (Ax2) cells (3). Briefly, exponentially growing *Dictyostelium* cells (100 µl at $5x10^6$ cells/ml) were harvested, washed in electroporation buffer (H50), and mixed with 6 µg plasmid, or 10 µg PCR product followed by exposure to two consecutive pulses of 0.65 kV (capacitance- 25 µF) in a chilled 1-mm gap electroporation cuvette (Sigma, Z706078). Transformed overexpressor cells were selected for with 10 µg/ml G418 (ptx-GFP) or hygromycin (InvivoGen, ant-hg-1) (pdm451).

RT-PCR to confirm knockout RNA was extracted from cells using the RNeasy kit (Qiagen, 74104). DNA was removed from the RNA using the DNA-free™ DNA removal kit (Thermo Fisher Scientific, AM1906). cDNA was synthesised using the RevertAid First Strand cDNA

Synthesis Kit (Thermo Fisher Scientific, K1622). PCR amplification of the knockout gene and Ig7 (positive control, DDB_G0294034) was carried out.

Western blotting for p-4E-BP1, total 4E-BP1, p-ATK-substrate, p-AKT(Thr308), p-AKT(Ser473), PKBR1 (T309) and PKBA (T278) (using p-PKC (pan)), p-AMPK, pS6K and total-S6K The p-PKC (pan) antibody was used in *Dictyostelium* to detect the phosphorylation state of the activation loops of PKBR1 (T309) and PKBA (T278) in chemotactically competent cells as described in Kamimura et al, 2019 (4). For all other *Dictyostelium* samples whole cell lysates were prepared by lysing cells directly into 2x laemmli (7.5 x 10^7 cells/ml)) boiling at 98°C for 6.5 mins and loading 6 µl (equivalent to 30 µg). Alternatively, homogenised rat hippocampal slices and patient derived astrocytes were mixed 1:1 with 2x laemmli and centrifuged at max speed for 10 mins at 4ºC before loading 15 µg or 7.5 µg respectively, without boiling. Samples were fractioned using a 15% gel for p-4E-BP1, total 4E-BP1 and p-AKT-substrate and a 10% gel for p-AMPK, total-AKT, p-AKT(Ser473), p-AKT(Thr308), p-S6K, total-S6K, and p-PKC-pan. Proteins were transferred to a 0.2 µm polyvinylidene difluoride membrane (Thermo Fisher Scientific, 88520) (p-4E-BP1 and total 4E-BP1), a 0.45 µm polyvinylidene difluoride (Millipore, IPFL00010) (p-AKT-substrate, p-PKC (pan)), or a 0.2 µm nitrocellulose membrane (Thermo Fisher Scientific, 15249794) (p-AMPK, total-AKT, p-AKT(Ser471), p-AKT(Thr308), p-S6K and total-S6K) by wet transfer at 15 Volts overnight (p-4E-BP1 and total 4E-BP1) or 90 Volts for 60 minutes (p-AKT substrate, p-AMPK, total-AKT, p-AKT(Ser471), p-AKT(Thr308), p-S6K, total-S6K and p-PKC (pan)) (Bio-Rad, UK). Membranes were incubated for 10 seconds with ponceau S solution before destaining in TBST. Membranes were blocked with 5% milk in TBS, 5% BSA in TBS, or odyssey blocking buffer (LI-COR 927-50100) for 1 hour. Following blocking the membranes were incubated in 5% milk in TBST, 5% BSA in TBST or odyssey blocking buffer (with 1:1000 Tween®20) with antibodies against p-4E-BP1 (1:1000 p-4E-BP1(Thr37/46) rabbit antibody, cell signalling technology, 9459S), total 4E-BP1 (1:1000 non-phospho-4E-BP1 (Thr46) rabbit antibody, cell signalling technology, 4923), p-AKT substrate (1:1000 Phospho-Akt Substrate rabbit antibody, cell signalling technology 10001), p-AMPK (1:2000 Phospho-AMPKα (Thr172) (40H9) rabbit antibody, cell signalling technology 2535), p-S6K (1:1000, Phospho-p70 S6 Kinase (Thr421/Ser424) rabbit antibody, 9204), total-S6K (1:1000, p70 S6 Kinase rabbit antibody, 9202), p-AKT(Ser473) (1:2000 Phospho-AKT (Ser473) rabbit antibody, cell signalling technology, 4060), p-AKT(Thr308) (1:1000 Phospho-AKT (Thr308) rabbit antibody, cell signalling technology, 9275), total-AKT (1:5000 AKT rabbit antibody, cell signalling technology 9272), or p-PKC (pan) (1:2000 Phospho-PKC (pan) (zeta Thr410) rabbit antibody, cell signalling technology 2060) at 4 °C overnight. Streptavidin (1:5000 Streptavidin, Alexa Fluor™ 680 conjugate, Thermo Fisher, S21378) was used as a loading control for *Dictyostelium* samples, and β-Actin (Sigma, A1978) was used as a loading control for rat hippocampal samples and astrocytes. Membranes were washed in TBST and incubated with a 1:2000 dilution of polyclonal goat anti-rabbit immunoglobulins/HRP (Agilent Technologies, DAKO P0448) (for p-4E-BP1, total 4E-BP1, and p-S6K) or a 1:10000 dilution of IRDye® 800CW goat anti-rabbit IgG secondary antibody (LI-COR, 925-3221) (for p-AKT-substrate, total-AKT, p-AKT(Ser471), p-AKT(Thr308), total-S6K, p-AMPK and p-PKC (pan)), and a 1:10000 Goat anti-mouse antibody (Cell signalling technology, 5470) (for β-Actin) in 5% milk in TBST, 5% BSA in TBST or odyssey blocking buffer (with 1:1000 Tween®20) for 1h. Blots were washed in TBST and visualised using the Odyssey CLx imager (LI-COR). For p-4E-BP1, total 4E-BP1, and p-S6K Odyssey chemifluorescent substrate kit (Li-Cor Biosciences, Odyssey®Chemifluorescent Substrate Kit 928-30005) was used. Blots were quantified using LI-COR Image Studio™, and normalised to the loading control, or total (unphosphorylated) protein (as specified).

Western blotting for GFP and RFP Whole cell lysates were prepared by lysing cells in RIPA buffer ($5x10^7$ cells/ml) containing protease inhibitor (Roche, 04693159001). Lysates were boiled in loading buffer and fractioned using a 12.5% gel, and transferred to a PVDF membrane (Millipore, IPFL00010) by wet transfer at 90 Volts for 60 minutes (Bio-Rad, UK). After incubation with 5% BSA in PBS for 60 min, the membrane was incubated in 5% BSA in PBS with antibodies against GFP (Chromotek, 3H9, 1:1000) or RFP (Chromotek, 5F8, 1:1000) at 4 °C overnight. Membranes were washed in PBST and incubated with a 1:10000 dilution of odyssey goat anti-rat IR DYE 800 (Li-Cor Biosciences, UK), and 1 in 10000 streptavidin (Thermo Fisher Scientific, UK) in 5% BSA in PBS for 1hour. Blots were washed in PBST and visualised using the Odyssey CLx imager (LI-COR).

Immunoprecipitation GFP-Trap®A beads were used for Immunoprecipitation of GFP-fusion proteins (Chromotek, gta). Briefly, *Dictyostelium* cells (1×10^7) co-expressing the indicated GFP- and the RFP-tagged proteins were pelleted and washed in phosphate buffer before being suspended in GFP-trap Lysis buffer. The extract was centrifuged, and the pellet discarded. The lysate was diluted with dilution buffer and incubated for 1 hour at 4°C with GFP-trap beads (Chromotek, gta). The beads were washed with dilution buffer, suspended in laemmli and boiled at 100°C for 5 min before running on an SDS-PAGE gel for analysis.

ATPase assay *Dictyostelium* cells (wild type or *UBXD18*$^-$ expressing Ddp97-RFP) in the exponential phase of growth were treated for 24 hours with compound (60 µM decanoic acid, 120 µM octanoic acid or 7.5 µM p97 inhibitor DBeQ (Tocris Bioscience, 4417). RFP-Trap®A beads were used for the immunoprecipitation of RFP tagged proteins (Ddp97-RFP) (Chromotek, rta). Following immunoprecipitation the RFP-Trap®A beads were washed with dilution buffer, and suspended in 10 µl HNG buffer (20 mM HEPES pH 8.5 (Sigma, H3375), 13 mM NaCl (Sigma, S9888), 1% glycerol (Sigma, G2025)), and used for ATPase activity analysis using the malachite green phosphate assay kit (Sigma, MAK307) according to published methodology (Rule et al., 2016) and manufacturer's procedure. Briefly, ATP hydrolysis reactions were set up using 5 µl of RFP-Trap®A beads, 6 µl of 5X HNG buffer, 1.5 µl of $MgCl_2$ (100 µM) (Sigma, M8266) and 1.5 µl of ATP (100 µM) (Sigma, A2383), and 16 µl H_2O. The reaction was incubated at 37 °C for one hour with 5 µl aliquots being removed at 15-minute intervals and diluted 1:50 in HNG buffer before freezing on dry ice. Free phosphate concentrations were assessed using the malachite green phosphate assay kit (Sigma, MAK307). Phosphate concentrations were standardised to protein concentration measured directly on the beads using a Bradford assay.

Fluorescence microscopy Ptx-GFP-*ubxd*18 or ptx-GFP-empty plasmids were electroporated into cells (3). Cells expressing the plasmids where imaged under 1% agar (Sigma, A5306) using an Olympus IX71 wide-field fluorescence microscope. Images were captured using a QICAM FAST 1394 camera.

Rat Hippocampal slices preparation for western blot ~270g Adult Sprague Dawley rats (males and females) were deeply anesthetised with 2-3% isoflurane in oxygen (2 L/min) and once unresponsive, the animal was decapitated. The brain was removed and placed in a glass petri dish filled with bubbled ice cold artificial cerebrospinal fluid (aCSF) (125 mM NaCl, 26 mM $NHCO_3$, 15 mM Glucose, 1.25 mM NaH_2PO_4, 2.5 mM KCl, 1.3mM $MgSO_4$ 7 H_2O, 2 mM $CaCl_2$, pH 7.4 and osmolality 305-310 mOsm). Coronal slices (350 µm) were sliced with a Leica VT1200 vibrating blade microtome. Hippocampal slices were transferred to submerged chambers (95% O_2, 5% CO_2) containing either aCSF only or aCSF with solvent control (DMSO), 100 µM or 300 µM Decanoic acid (Sigma Aldrich, C1875) in DMSO. The chambers were placed into a 34°C bath for 20 minutes and then moved at room temperature for 40 min in

carbonated standard recording cerebrospinal fluid. Samples were snap frozen in liquid nitrogen and lysed in a mixture of RIPA buffer (150 mM sodium chloride, 1.0% Triton X-100, 0.5% sodium deoxycholate, 0.1% sodium dodecyl sulfate, 50 mM Tris, pH 8.0. Sigma Aldrich, R0278) and protease inhibitors (Thermo Fisher, A32963). The tissue was mechanically disrupted by a mechanical rotor type homogenizer (FastPrep-24, MP Biomedicals LLC) with the use of homogenizer beads (SLS Scientific Laboratory Supplies, D1031-01). The samples were then centrifuged at max speed for 10 minutes, the pellet discarded and stored at -80°C. Protein concentration was determined by Bradford assay, samples were mixed 1:1 with 2x laemmli, and 15 µg of protein was analysed by western blot. A total of 6 rats were analysed, with 3 coronal slices assessed for each treatment condition per rat.

Patient derived astrocyte differentiation, and preparation for western blot iPSCs from three individual control patients (hVS-228, hVS-420, and hVS-421), two patients with TSC1 mutations (hVS233, and hVS-401), and two patients with TSC2 mutations (hVS-417, and hVS-424), were differentiated into astrocytes as described in Nadadhur et al. (2018) (5). At day 45 of the differentiation protocol cells were switched to Sanbio medium to form a pure astrocyte culture, and maintained until day 76. At day 76 astrocytes were plated on geltrex coated 12 well plates in Sanbio astrocyte medium, and allowed to recover from plating. At day 80, half of the medium was refreshed with Sanbio astrocyte medium containing either decanoic acid (final concentration in well: 300 µM) or DMSO. After 24h, cells were harvested by removing medium, adding 50 µl lysis buffer (50 mM HEPES, 150mM NaCl, 1mM EDTA, 2.5 mM EGTA, 0.1% Triton X-100, 10% glycerol, 1mM DTT, 1x protease inhibitor, 1x phosphatase inhibitor) per well on ice and scraping cells off the plate. Cells from each well were collected in a tube and frozen (n=10 per patient). Protein concentration was determined by Bradford assay, samples were mixed 1:1 with 2x laemmli and 7.5 µg of protein was analysed by western blot.

Statistics The distribution of all experimental data was tested using the D'Agostino and Pearson omnibus normality test. Independent data showing a Gaussian distribution were analysed using parametric tests. Normally distributed data from two groups were statistically analysed using two-tailed unpaired t-tests. The one-way ANOVA statistical test was used to test for significance between the means of three or more independent groups of normally distributed data, in conjunction with Dunnett's post hoc test. Two-tailed non-parametric Mann-Whitney t-tests were used to assess significance between independent samples from two groups not showing a Gaussian distribution. The Kruskal-Wallis test with Dunn's post hoc test was used to test significance between three or more independent groups of data not showing a Gaussian distribution. Western blot data from patient derived astrocytes showed a Gaussian distribution and were analysed using nested t-tests to account for a lack of independence between astrocytes from individual patients cultured in separate wells. Statistical analysis was carried out using GraphPad Prism Software. Where no significance is indicated in a figure, $p > 0.05$.

References

1. A. R. Hussain *et al.*, Dual Targeting of mTOR Activity with Torin2 Potentiates Anticancer Effects of Cisplatin in Epithelial Ovarian Cancer. *Mol Med* **21**, 466-478 (2015).
2. R. Sekine, T. Kawata, T. Muramoto, CRISPR/Cas9 mediated targeting of multiple genes in Dictyostelium. *Scientific Reports* **8**, 8471 (2018).
3. P. Gaudet, K. E. Pilcher, P. Fey, R. L. Chisholm, Transformation of Dictyostelium discoideum with plasmid DNA. *Nature Protocols* **2**, 1317 (2007).

4. Y. Kamimura, M. Tang, P. Devreotes, Assays for chemotaxis and chemoattractant-stimulated TorC2 activation and PKB substrate phosphorylation in Dictyostelium. *Methods Mol Biol* **571**, 255-270 (2009).
5. A. G. Nadadhur *et al.*, Patterning factors during neural progenitor induction determine regional identity and differentiation potential in vitro. *Stem Cell Res* **32**, 25-34 (2018).
